# Discovery, Herbicidal Activity and Biosynthesis of a Novel Natural Tetramic Acid from *Alternaria Species*

**DOI:** 10.1002/advs.202416188

**Published:** 2025-04-25

**Authors:** He Wang, Yanjing Guo, Qing Liu, Jing Zhang, Qianlong Zhang, Mingying Yang, Qizhen Chen, Sheng Qiang, Bernal E. Valverde, Shiguo Chen

**Affiliations:** ^1^ State Key Laboratory of Agricultural and Forestry Biosecurity College of Life Sciences Nanjing Agricultural University Nanjing 210095 P. R. China; ^2^ Research and Development in Tropical Agriculture Alajuela 4050 Costa Rica

**Keywords:** bioherbicide, biosynthetic pathway, natural product, structure‐activity relationship, tetramic acid

## Abstract

The tetramic acid moiety is a pivotal structural unit in numerous natural products. As an analogue of the simplest tetramic acid compound tenuazonic acid (TeA), sec‐pentyl‐TeA (S‐TeA) exhibits double herbicidal activity of TeA. Here, this work identifies S‐TeA as a novel natural product synthesized by *Alternaria alternata* and three other filamentous fungi. Chiral analysis confirm the absolute configuration of natural S‐TeA as (5*S*, 6*S*). Configuration‐bioactivity studies reveal that natural (5*S*, 6*S*)‐S‐TeA is the eutomer and possesses the highest herbicidal activity among all tested diastereomers. Biosynthetic analyses demonstrate that threonine is the precursor to S‐TeA, beginning with the production of 2‐amino‐3‐methylhexanoic acid (AMHA) via eight enzymes from the branched‐chain amino acid (BCAA) biosynthetic pathway, including threonine deaminase, 2‐isopropylmalate synthase (IPMS), 3‐isopropylmalate dehydratase (IPMDH), isopropylmalate dehydrogenase (ISMD), acetolactate synthase, ketol‐acid reductoisomerase, dihydroxy acid dehydratase, and BCAA aminotransferase. Subsequently, AMHA undergoes acetylation and cyclization by non‐ribosomal peptide synthetases to form S‐TeA. Distinct differences in the biosynthetic pathways of S‐TeA and TeA are identified. In vitro studies confirm the critical roles of three unique enzymes IPMS, IPMDH, and ISMD in S‐TeA biosynthesis, which are absent in TeA biosynthesis. These findings provide a solid basis for developing S‐TeA as a natural product herbicide.

## Graphical abstract

The biosynthetic mechanism of novel natural tetramic acid S‐TeA in *Alternaria alternata* is elucidated. Threonine acts as a precursor for 2‐amino‐3‐methylhexanoic acid (AMHA) synthesis via eight key enzymes from the branched‐chain amino acid biosynthetic pathway in the mitochondrion, wherein three unique enzymes (IPMS, IPMDH, and ISMD) play critical roles. Subsequently, AMHA undergoes acetylation and cyclization by nonribosomal peptide synthetases (NRPS) to form S‐TeA in the cytosol.

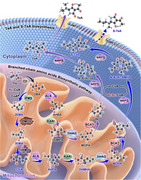

## 1 Introduction

Over the past 70 years, synthetic herbicides have been instrumental in modernizing agriculture due to their efficiency and economic benefits for farmers. However, the prolonged and heavy reliance on conventional synthetic herbicides has raised concerns about the increasing evolution of herbicide‐resistant weeds, environmental contamination, and risks to human health.^[^ ^1^, ^2^ ^]^ There is an urgent need to develop new herbicides with novel target sites and safer environmental and toxicological profiles.^[^ ^3^, ^4^ ^]^

Natural products are widely considered a valuable and sustainable resource for discovering new structural leads and molecular targets for pesticides due to their excellent bioactivity, diverse structures, unique targets, and lower environmental impact.^[^ ^5^ ^]^ Among natural products, the tetramic acid moiety (2,4‐pyrrolidinedione) is a crucial structural component. This moiety is a prominent feature in many natural products and has attracted the attention of pharmacologists, agriculturalists, biologists, and chemists because of its wide‐ranging bioactivities and diverse molecular architectures.^[^ ^6^, ^7^ ^]^ Several tetramic acids are known for their antifungal, antibacterial, antiviral, anticancer, antioxidant, and herbicidal activities; with some displaying multiple biological effects.^[^ ^8^, ^9^, ^10^ ^]^ Tenuazonic acid (TeA) is a well‐known 3‐acyltetramic acid compound that can be produced by various phytopathogenic fungi, particularly *Alternaria* species.^[^ ^11^ ^]^ Previous studies demonstrated that TeA is a promising natural photosystem II (PSII) inhibitory herbicide, as it disrupts PSII electron transport by binding to the D1 protein, showing broad spectrum, rapid, and high herbicidal activity.^[^ ^12^ ^]^ Its pyrrole ring, containing an acylamide group, is essential for photosynthetic inhibitory activity, which can also be influenced by the length of the alkyl side chain at the 5‐position.^[^ ^12^, ^13^ ^]^ A novel derivative, 3‐acetyl‐5‐sec‐pentyltetramic acid (named sec‐pentyl‐TeA, S‐TeA, CAS Registry No. 2385071‐63‐4), was identified among a series of TeA's derivatives synthesized and tested for herbicidal activity, displaying twice the photosynthetic inhibitory capacity and herbicidal efficacy of natural TeA.^[^ ^14^ ^]^ This compound has been patented in the US and Europe and currently under commercial registration in China.^[^ ^15^, ^16^ ^]^ Since *A. tenuis* can produce 3‐acetyl‐5‐isopropyltetramic acid and 3‐acetyl‐5‐isobutyltetramic acid using *L*‐valine and *L*‐leucine as precursors, respectively,^[^ ^17^ ^]^ it is likely that *Alternaria* species might also be capable to producing S‐TeA. To date, no reports confirm the natural occurrence of S‐TeA, but if confirmed, it would enhance its potential as a bioherbicide.

While many natural pesticides exhibit satisfactory bioactivity both in laboratory and field trials, their low abundance in source organisms limits industrial production and commercial success.^[^ ^18^, ^19^ ^]^ Advances in genetic and metabolic engineering and synthetic biology provide a promising strategy for large‐scale production of natural products through microbial cell factories.^[^ ^20^, ^21^, ^22^ ^]^ Identifying the biosynthetic pathways and regulatory genes of natural products in their native organisms is essential for successful biomanufacturing. Thanks to advances in genomics, metabolomics, and bioinformatics, several biosynthetic gene clusters for tetramic acid production have been elucidated.^[^ ^23^ ^]^ Current evidence suggests that many 3‐acyltetramic acids are hybrid natural products synthesized from polyketide and *α*‐amino acid precursors via polyketide synthases (PKSs) and non‐ribosomal peptide synthetases (NRPSs).^[^ ^7^, ^8^ ^]^ However, further research is needed to identify the key genes and complete the biosynthetic pathway of S‐TeA in various fungal species.

This study hypothesized that S‐TeA is a new, naturally occurring secondary metabolite. A compound from *Alternaria alternata* fermentation broth with retention time similar to synthetic S‐TeA was isolated and purified using medium‐pressure liquid chromatography (MPLC). The structure was confirmed using mass spectrometry (MS), infrared spectroscopy (IR), and nuclear magnetic resonance (NMR) analyses, verifying its identity as S‐TeA. The compound was also detected in the fermentation broth of the filamentous fungus *Magnaporthe oryzae*, *A. altern*ata f. sp. *lycopersici*, and *A. brassicicola*, indicating that S‐TeA is indeed a naturally occurring metabolite. The absolute configuration of natural S‐TeA was further analyzed using chiral high performance liquid chromatography (Chiral‐HPLC) and electron circular dichroism (ECD). Structure‐activity relationships were explored by evaluating the binding affinity to the D1 protein, photosynthetic inhibitory activity and herbicidal efficacy of synthetic S‐TeA compounds with different configurations. The biosynthetic pathway and regulatory mechanisms of S‐TeA production in *A. alternata* were investigated using transcriptomics, untargeted metabolomics, and precursor‐feeding experiments. An isotope tracing technique verified key steps in the S‐TeA biosynthetic pathway, and the functions of three critical enzymes encoded by genes specific to the S‐TeA biosynthetic pathway were characterized in *vitro*. This study provides both a solid basis for developing S‐TeA as a natural product herbicide and fundamental insights for environmentally friendly, large‐scale biomanufacturing of high‐activity S‐TeA.

## 2 Results and Discussion

### 2.1 Isolation, Purification and Identification of Natural S‐TeA Production in *Alternaria* and Other Filamentous Fungi

**Figure 1:**
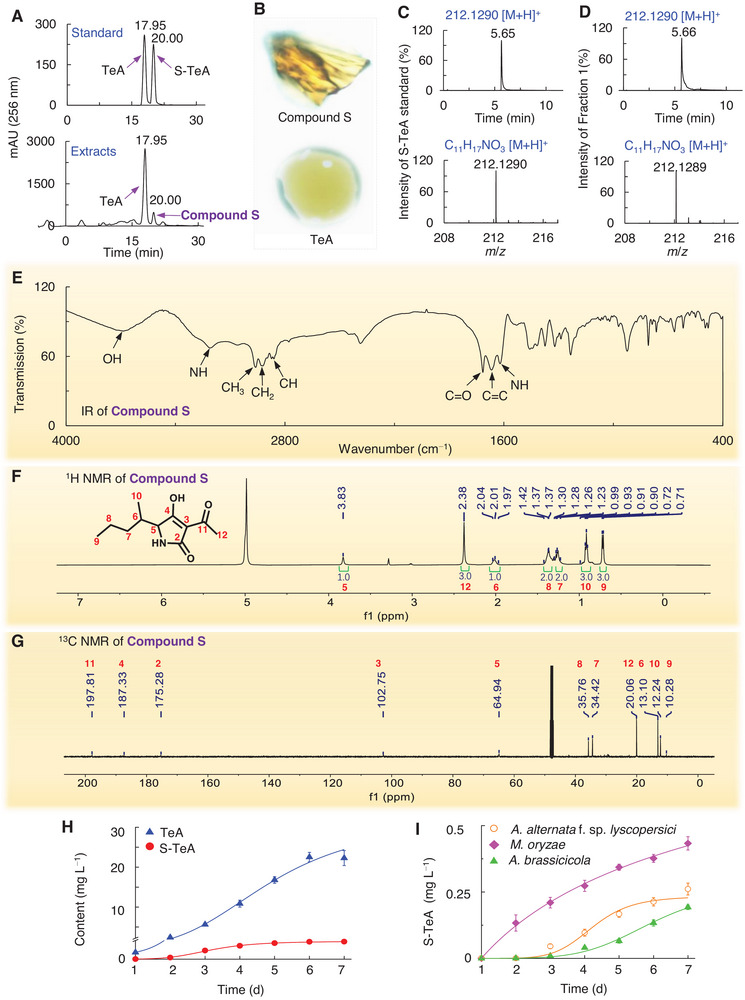
Identification of the chemical structure of S‐TeA purified from *A. alternata* and quantification of S‐TeA produced by different filamentous fungi. A) MPLC chromatogram of standard solutions of TeA and S‐TeA (above), and the crude extract from the fermentation broth of *A. alternata* (below). The peaks of the TeA and S‐TeA of standards and those from the crude extract corresponding to TeA and of designated Compound S, that has the same retention time as the standard S‐TeA, are labelled. B) The purified Compound S and TeA at room temperature. C) The UPLC‐MS chromatogram and mass spectrum in positive ion mode of S‐TeA standard. D) The UPLC‐MS chromatogram and mass spectrum in positive ion mode of Compound S. E) IR spectrum of Compound S. F) ^1^H NMR spectrum (500 MHz, methanol‐*d_4_*) of Compound S. G) ^13^C NMR spectrum (125 MHz, methanol‐*d_4_*) of Compound S. H) S‐TeA and TeA production of *A. alternata* during the first 7 days of culture on Czapek liquid medium. Data shown are mean values ± SD of three independent biological replicates. I) S‐TeA production of three fungi during the first 7 days of culture on Czapek liquid medium. Data are presented as mean values ± SD of three independent biological replicates.

To determine if S‐TeA is naturally produced, metabolites obtained from an *A. alternata* fermentation broth, along with synthetic TeA and S‐TeA standards, were analyzed using MPLC. The chromatogram for the TeA and S‐TeA standards displayed distinct peaks at retention times of 18 and 20 min, respectively (**Figure** **1**A). In the chromatographic profile of the fermentation broth extracts, a distinct peak labeled as Compound S appeared at 20 min, matching the retention time of the S‐TeA standard (Figure 1A). This suggests that *A. alternata* may naturally produce free S‐TeA. Subsequently, *A. alternata* was cultivated on a larger scale in Czapek liquid medium to obtain sufficient material, allowing for the collection and purification of the fractions for TeA and Compound S at 18 and 20 min using MPLC for purification and crystallization. TeA appeared as a light brown oil while Compound S crystalized as an orange solid at room temperature (Figure 1B).

The structure of Compound S was confirmed using MS, IR, and NMR. The LC‐MS spectrum data had a prominent parent ion peak at *m/z* 212.1289 ([M+H]^+^) with a retention time of 5.66 min in the positive mode, consistent with the synthetic S‐TeA standard (Retention time = 5.66 min, *m/z* 212.1290 [M+H]^+^) (Figure 1C,D). The IR spectrum was analyzed to identify functional groups (Figure 1E). A peak at 3674 cm^−1^ was attributed to the stretching vibration of a hydroxy group. Absorption peaks at 3200 and 1617 cm^−1^ correspond to the stretching and asymmetric bending vibrations of a secondary amine group, respectively. Three peaks between 2959 and 2860 cm^−1^ indicated the presence of alkyl groups in Compound S. The characteristic peak at 1713 cm^−1^ corresponds to the carbonyl stretching vibration in the lactam ring, while a peak at 1665 cm^−1^ was associated with the stretching vibration of a carbon‐carbon double bond. These results are consistent with established IR data for tetramic acid compounds.^[^ ^24^ ^]^

Further structural confirmation was obtained through detailed NMR analysis. The ^1^H NMR spectrum of Compound S in methanol‐*d*_4_ displayed signals for an amide‐bearing methine (*δ* 3.83, H‐5), a carbonyl‐bearing methyl (*δ* 2.38, H‐12), one methine (*δ* 2.01, H‐6), two methylene groups (*δ* 1.37, H‐8; *δ* 1.26, H‐7), and two methyl groups (*δ* 0.91, H‐10; *δ* 0.72, H‐9) (Figure 1F). Additionally, the ^13^C NMR spectrum of Compound S indicated the presence of two carbonyl groups (*δ* 197.81, C‐11; *δ* 175.28, C‐2), one enolic carbon (*δ* 187.33, C‐4), and one olefinic quaternary carbon (*δ* 102.75, C‐3) (Figure 1G). Analysis of ^1^H‐^1^H correlated spectroscopy (COSY) cross‐peaks revealed a sec‐pentyl moiety and a carbonyl‐bearing methyl in Compound S (Figure S1A, Supporting Information). Heteronuclear multiple bond correlation (HMBC) cross‐peaks showed a crucial correlation between H‐5 (*δ* 3.83) and carbonyl carbon (*δ* 175.28), confirming the presence of a pyrrolidine structure. Additional HMBC correlations, such as between H‐12 (*δ* 2.38) and C‐3 (*δ* 102.75), and the correlation between H‐10 (*δ* 0.91) and C‐5 (*δ* 64.94), indicated that the sec‐pentyl moiety and acetyl group are connected to the 5‐ and 3‐positions of the pyrroline, respectively (Figure S1B, Supporting Information). Thus, Compound S was identified as 3‐acetyl‐4‐hydroxy‐5‐sec‐pentyl‐pyrrolidine‐2‐one (S‐TeA), matching the synthesized compound.^[^ ^14^ ^]^

To confirm that S‐TeA is a naturally occurring product, fermentation broth extracts of *A. alternata* cultivated for 1 to 7 days (d) in Czapek liquid medium were analyzed to monitor the dynamics of S‐TeA production using ultra‐performance LC‐MS spectrometry. Production of S‐TeA was detected on the second day, sharply increasing until the fifth day, and plateauing on the sixth day. The highest observed concentration of free S‐TeA reached 1.5 mg L^−1^ (Figure 1H). TeA displayed a similar accumulation pattern, but its content was consistently higher, reaching 20 mg L^−1^ on the sixth day.

Moreover, the fermentation broths of three other TeA‐producing fungi, *M. oryzae*, *A. alternata* f. sp. *lycopersici* and *A. brassicicola*, also cultivated for 1 to 7 days in Czapek medium, were analyzed to detect S‐TeA via LC‐MS.^[^ ^25^, ^26^ ^]^ As expected, S‐TeA (*m*/*z* 212.1287) was detected in all extracts, with retention times of 5.66 min for *M. oryzae*, 5.65 min for both *A. alternata* f. sp. *lycopersici* and *A. brassicicola* (Figure S2, Supporting Information). However, the signal intensity was distinctly lower compared to *A. alternata*. After 7 d, S‐TeA concentrations in *M. oryzae*, *A. alternata* f. sp. *lycopersici* and *A. brassicicola* were 0.43, 0.26 and 0.19 mg L^−1^, respectively (Figure 1I). This suggests that TeA producer fungi may also produce S‐TeA.

### 2.2 Validation of Absolute Configuration of Natural S‐TeA

#### 2.2.1 Phytotoxicity and Configuration Differences between Natural S‐TeA and Synthetic S‐TeA

**Figure 2:**
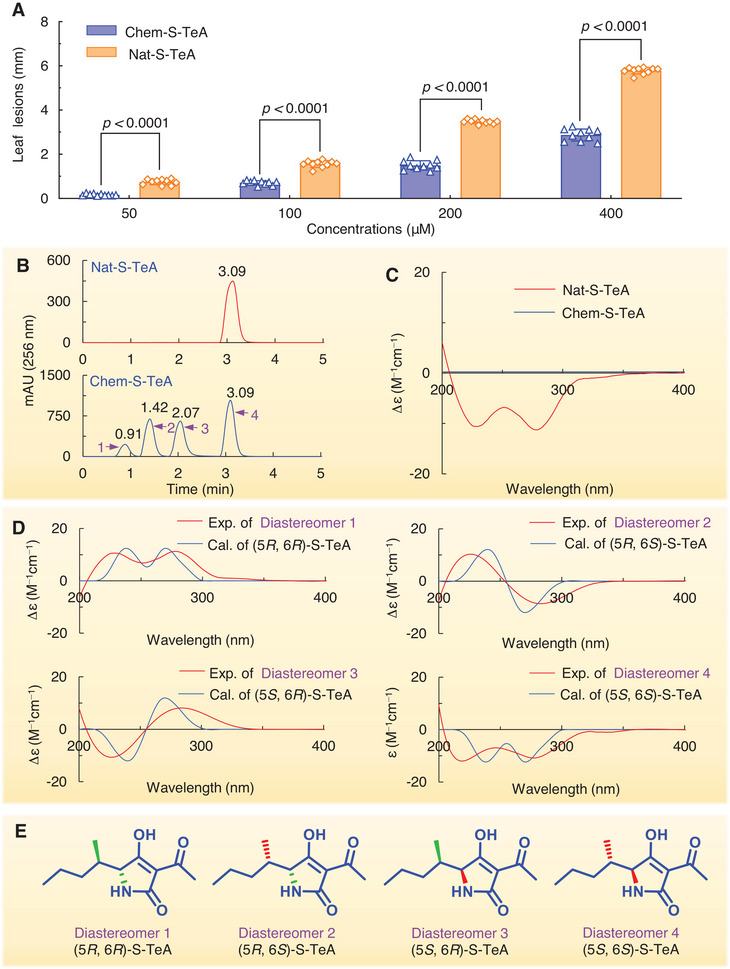
Phytotoxicity of natural S‐TeA (Nat‐S‐TeA) and chemical synthetic S‐TeA (Chem‐S‐TeA) to *A. adenophora*, and the identification of the absolute configuration of natural S‐TeA. A) Diameter of necrotic lesions of *A. adenophora* treated with Nat‐S‐TeA and Chem‐S‐TeA at increasing concentrations. Data shown are mean values ± SD of ten independent biological replicates. Significant differences are shown comparing the natural and chemicals forms of S‐TeA at each concentration by a Student's *t*‐test. B) HPLC chromatograms of Nat‐S‐TeA (above) and Chem‐S‐TeA (below) depicting diastereomers corresponding to isomeric forms given in E. C) ECD spectra of Nat‐S‐TeA and Chem‐S‐TeA. D) experimental (Exp.) ECD spectra of diastereomers 1 to 4 of Chem‐S‐TeA purified by chiral HPLC and computational (Cal.) ECD spectra of (5*R*, 6*R*)‐S‐TeA, (5*R*, 6*S*)‐S‐TeA, (5*S*, 6*R*)‐S‐TeA and (5*S*, 6*S*)‐S‐TeA, respectively. E) The stereoisomeric structures of Chem‐S‐TeA.

Over 80% of biologically active natural compounds are chiral, often containing multiple stereogenic elements. Typically, only the eutomer exhibits the desired biological effect, while the other enantiomer may be inactive or has adverse effects.^[^ ^27^, ^28^ ^]^ The absolute configuration of natural TeA was established as (5*S*, 6*S*) by ozonolysis and acidic hydrolysis, with only this form exhibiting biological activity while its isomer, allo‐TeA was inert.^[^ ^29^, ^30^ ^]^ From Figure 1F and S‐TeA contains two stereogenic centers. To evaluate the phytotoxicity of natural S‐TeA (Nat‐S‐TeA), lesions on mature leaves of *Ageratina adenophora* were monitored following treatment with Nat‐S‐TeA or artificial synthetic S‐TeA (Chem‐S‐TeA) at varying concentrations. Nat‐S‐TeA was found to be twice as phytotoxic as Chem‐S‐TeA on *A. adenophora* (**Figure** **2**A).

Chiral‐HPLC and ECD were used to investigate the configuration related phytotoxicity differences between Nat‐S‐TeA and Chem‐S‐TeA. The liquid chromatogram of Nat‐S‐TeA showed a single peak at 3.09 min, with 100% enantiomeric excess (ee%). In contrast, Chem‐S‐TeA showed four peaks 0.97, 1.28, 1.61 and 3.09 min in a 1:2:2:5 ratio, indicating a mixture of four diastereomers (Figure 2B). The ECD spectrum of Nat‐S‐TeA revealed two negative Cotton effects (CE) at 228 nm and 278 nm, absent in the Chem‐S‐TeA ECD spectrum (Figure 2C). The absence of a visible CE may be attributed to a low concentration (100 µM) of Chem‐S‐TeA used in the detection. Several studies have demonstrated a linear positive correlation between ECD signal intensity and the concentration of enantiomeric amino acid or diastereomeric *β*‐lactam mixtures.^[^ ^31^, ^32^ ^]^ These findings confirm that Nat‐S‐TeA is a single chiral entity, while Chem‐S‐TeA is a composition of four stereoisomers.

#### 2.2.2 Determining the Absolute Configuration of Natural S‐TeA

ECD is as a powerful tool for determining the absolute configuration of active compounds due to its low sample consumption and recyclability.^[^ ^27^, ^33^ ^]^ To confirm the absolute configuration of Nat‐S‐TeA, the four diastereomers of Chem‐S‐TeA were successfully separated via chiral HPLC, and their ECD spectra were recorded, computationally calculated, and compared. The overall experimental and calculated spectra showed excellent agreement (Figure 2D), confirming that the configuration of diastereomers 1 to 4 were (5*R*, 6*R*), (5*R*, 6*S*), (5*S*, 6*R*), (5*S*, 6*S*). The absolute configuration of Nat‐S‐TeA was determined to be (5*S*, 6*S*) (Figure 2E). Obviously, the phytotoxic activity of S‐TeA is strongly dependent on its configuration. Previous studies have demonstrated that natural (5*S*, 6*S*)‐TeA exhibits significantly higher bioactivity compared to other diastereomers and synthetic TeA with alternative configurations.^[^ ^14^, ^29^, ^30^ ^]^

#### 2.2.3 Relationship between Configuration and Bioactivity of S‐TeA

**Figure 3:**
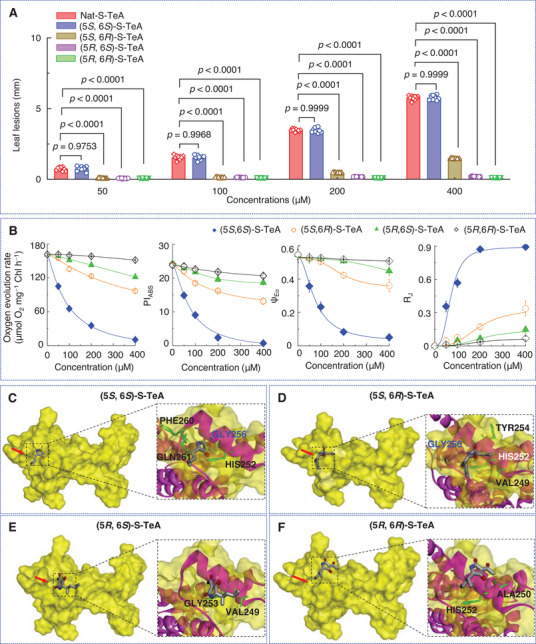
Phytotoxic and photosynthetic activity of individual S‐TeA diastereomers. A) Diameter of necrotic lesions of *A. adenophora* treated with Nat‐S‐TeA, (5*S*, 6*S*)‐S‐TeA, (5*S*, 6*R*)‐S‐TeA, (5*R*, 6*S*)‐S‐TeA or (5*R*, 6*R*)‐S‐TeA at increasing concentrations. Data shown are mean values ± SD of ten independent biological replicates. Significant differences shown compare Nat‐S‐TeA with each isomer at each concentration using a Student's *t*‐test. B) The concentration‐dependent effect of S‐TeA diastereomers on the rate of O_2_ evolution or fluorescence parameters PI_ABS_ (PSII performance index), ψ_Eo_ (probability that an electron moves further than Q_A_^−^) and R_J_ (number of PSII RCs with Q_B_‐site filled with compounds). Data shown are mean values ± SD. Each data point is the average of 30 measurements from three independent biological replicates. C‐F) Individual stereo views of (5*S*, 6*S*)‐S‐TeA, (5*S*, 6*R*)‐S‐TeA, (5*R*, 6*S*)‐S‐TeA and (5*R*, 6*R*)‐S‐TeA binding environment at the Q_B_ site.

The relationship between the configuration and phytotoxicity of S‐TeA was evaluated using mature *A. adenophora* leaves treated with Nat‐S‐TeA or synthetic S‐TeA in each of its four different configurations, across increasing doses. Leaf lesions caused by TeA, as well as by both Chem‐S‐TeA and Nat‐S‐TeA, became visually evident at concentrations of 100 µM and above, after 48 h post treatment. S‐ TeA, especially Nat‐S‐TeA, was more phytotoxic than TeA (Figure S3A,B, Supporting Information). The Nat‐S‐TeA and the synthetic (5*S*, 6*S*)‐S‐TeA isomer were the most active, as indicated by the earliness and severity of lesion development, with effects being concentration dependent (**Figures** **3**A, S3C,D, Supporting Information). The (5*S*, 6*R*)‐S‐TeA and (5*R*, 6*S*)‐S‐TeA diastereomers also induced lesions in a dose dependent manner, though their effects were less pronounced, becoming noticeable at 200 and 400 µM, respectively. The (5*R*, 6*R*)‐S‐TeA isomer was inactive (Figure 3A, Figure S3D, Supporting Information). These findings suggest that S‐TeA bioactivity is determined by configurations of C5 and C6 of the molecule. The interaction between components in the mixture of the diastereomers affects biological activity (synergistically, additively, or antagonistically), which must be determined experimentally.^[^ ^34^ ^]^ For S‐TeA, the 5*S* configuration is crucial for its activity, and the (5*S*, 6*S*) form of Nat‐S‐TeA explains its enhanced activity compared to Chem‐S‐TeA.

TeA and some of its analogues are photosynthetic inhibitors, with their core structure featuring the amide group in the pyrrole ring.^[^ ^12^ ^]^ Here, the (5*S*, 6*S*)‐S‐TeA caused a significant, concentration‐dependent decrease in the oxygen evolution rate, with a 50% inhibitory concentration (*I*_50_) value of 78 µM. The *I*_50_ values for (5*S*, 6*R*)‐S‐TeA, (5*R*, 6*S*)‐S‐TeA, and (5*R*, 6*R*)‐S‐TeA exceeded the highest treatment concentrations needed to achieve an equivalent inhibitory effect to (5*S*, 6*S*)‐S‐TeA (Figure 3B). This aligns with the pronounced concentration‐dependent impact of (5*S*, 6*S*)‐S‐TeA on the fast rise of the J‐step (at 2 ms) of the chlorophyll *a* fluorescence kinetics curve (Figure S4A, Supporting Information). At 200 µM, (5*S*, 6*S*)‐S‐TeA led to a more pronounced J‐step increase than the other three diastereomers (Figure S4, Supporting Information). Only minor J‐step rises in the fluorescence kinetics curves were observed at 200 µM for (5*S*, 6*R*)‐S‐TeA and at 400 µM for (5*R*, 6*S*)‐S‐TeA at (Figures S4B, C, Supporting Information). The (5*R*, 6*R*)‐S‐TeA isomer did not affect the J‐step rise even at the highest concentrations (Figure S4D, Supporting Information). A rapid J‐step increase is characteristic of the fluorescence rise kinetics of PSII inhibitory herbicide diuron (3‐(3, 4‐dichlorophenyl)‐1,1‐dimethylurea)‐ and TeA‐treated plants, linked to Q_A_ reduction in PSII reaction centers due to blocked electron flow beyond Q_A_.^[^ ^13^, ^35^ ^]^ Concomitantly, (5*S*, 6*S*)‐S‐TeA decreased the PSII performance index PI_ABS_ (expressing the overall activity of PSII) in a concentration‐dependent manner, to a greater extent than the other three diastereomers (Figure 3B). The *I*_50_ value for PI_ABS_ with (5*S*, 6*S*)‐S‐TeA was 79 µM, closely aligning with that for the PSII oxygen evolution rate. The *I*_50_ values for (5*S*, 6*R*)‐S‐TeA, (5*R*, 6*S*)‐S‐TeA and (5*R*, 6*R*)‐S‐TeA were similarly higher than the maximum treatment concentrations required to achieve an equivalent inhibitory effect to (5*S*, 6*S*)‐S‐TeA. A similar pattern was observed for the probability of a trapped exciton moving an electron into the electron transport chain beyond Q_A_ (ψ_Eo_).^[^ ^35^ ^]^ Thus, the PSII electron transport inhibition in S‐TeA treated leaves decreases sharply when the configuration shifts from *S* to *R*. These findings are further corroborated by a significant increase in the R_J_ parameter (Figure 3B) in plants treated with (5*S*, 6*S*)‐S‐TeA compared to other diastereomers, indicating a higher Q_B_ site occupancy by a PSII inhibitor.^[^ ^36^, ^37^ ^]^ This suggests that (5*S*, 6*S*)‐S‐TeA likely exhibits the greatest binding affinity to the Q_B_ site among the S‐TeA diastereomers.

#### 2.2.4 Molecular Docking Analysis of Configuration and Bioactivity

To further explore the relationship between S‐TeA configuration and bioactivity, the molecular solvent‐accessible surface area (Molecular‐SASA), of the four S‐TeA diastereomers was modeled for binding to the Q_B_ site of the D1 protein in *A. adenophora* using Discovery Studio. As detailed in Table S1, Supporting Information, (5*S*, 6*S*)‐S‐TeA had a higher Molecular‐SASA value than the other diastereomers; with value decreasing as the configuration shifted from *S* to *R*. Higher Molecular‐SASA values are typically associated with increased biological activity.^[^ ^38^, ^39^ ^]^

Molecular docking models revealed distinct interactions between the D1 protein and the four S‐TeA diastereomers (Figure 3C‐F). Hydrogen bond was the primary force stabilizing the ligand−receptor complexes. For (5*S*, 6*S*)‐S‐TeA, D1‐Gly256 formed a key hydrogen bond with the carbonyl oxygen (O2), with a bond distance of 3.09 Å. Additionally, 11 residues, including D1‐His252, D1‐Gly253, D1‐Phe255, D1‐Arg257, D1‐Ile259, D1‐Phe260, D1‐Gln261, D1‐Tyr262, D1‐Ala263, D1‐Ser264, and D1‐Asn266 formed hydrophobic or van der Waals interactions with the alkyl side chain or acetyl group of (5*S*, 6*S*)‐S‐TeA, enhancing and stabilizing the complex, thus explaining its high binding affinity of −34.06 kcal mol^−1^ (Figure 3C, Figure S5A; Table S1, Supporting Information). (5*S*, 6*R*)‐S‐TeA, exhibited a similar hydrogen bond with D1‐Gly256, but with a longer distance (3.72 Å). Ten residues, eight in common with (5*S*, 6*S*)‐S‐TeA, formed alkyl hydrophobic or van der Waals interactions with (5*S*, 6*R*)‐S‐TeA. The lower binding affinity of (5*S*, 6*R*)‐S‐TeA to its target site compared to (5*S*, 6*S*)‐S‐TeA can be attributed to the nearly 1.20‐fold longer of hydrogen bond distance, 1.24‐fold higher of interaction energy, and less and different amino acid residues implicated in hydrophobic interactions (Figure 3D, Figure S5B; Table S1, Supporting Information). The (5*R*, 6*S*)‐S‐TeA and (5*R*, 6*R*)‐S‐TeA diastereomers did not form hydrogen bonds with the D1 protein, and exhibited fewer van der Waals interactions (Figure 3E, F, Figure S5C,D, Supporting Information). These differences contribute the sharp increase in binding interaction energy of (5*R*, 6*S*)‐S‐TeA and (5*R*, 6*R*)‐S‐TeA at the Q_B_ site, which were −15.23 and −8.66 kcal mol^−1^, respectively, compared with (5*S*, 6*S*)‐S‐TeA (Table S1, Supporting Information).

Stereo views of the docking models showed that the (5*S*, 6*S*)‐S‐TeA pyrrole ring entered deeply into Q_B_ pocket, while its sec‐pentyl alkyl side chain extended along the cavity wall, partially exposed (Figure 3C). Conversely, (5*S*, 6*R*)‐TeA partially entered the cavity, while both (5*R*, 6*S*)‐TeA and (5*R*, 6*R*)‐TeA were fully exposed outside the Q_B_ pocket (Figure 3D‐F). The molecular physicochemical properties of a compound dictate its target protein binding affinity.^[^ ^40^ ^]^ These data indicate a negative relationship between Molecular‐SASA values and hydrogen bond distance, and a positive relationship between Molecular‐SASA values and binding affinity. The *S* configuration at the 5‐position is crucial for orienting S‐TeA toward D1‐Gly256 to form a hydrogen bond, while the *S* configuration at the 6‐position optimized binding to the Q_B_ site. This explains why the photosynthetic inhibitory activity and phytotoxicity of (5*S*, 6*S*)‐S‐TeA surpass those of other diastereomers. This is also the fundamental cause that natural (5*S*, 6*S*)‐S‐TeA displays twice the herbicidal activity of synthetic S‐TeA with alternative configurations. These results underscore the importance of a chiral compound's configuration in determining target binding affinity.

### 2.3 Identification of the Precursor of S‐TeA Biosynthesis in *A. alternata*

**Figure 4:**
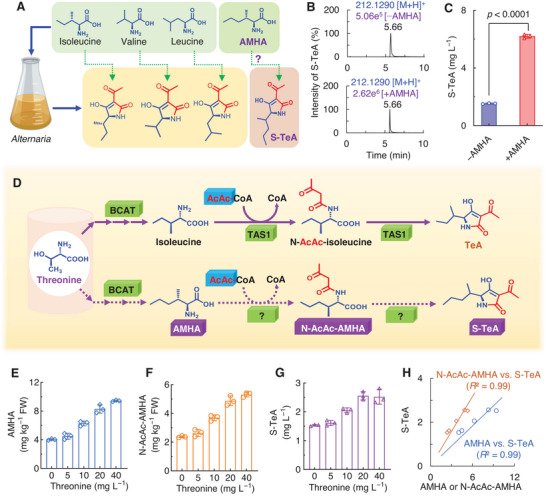
Verification of the precursor of S‐TeA biosynthesis in *A. alternata*. A) 2‐amino‐3‐methylhexanoic acid (AMHA) was assumed as a biosynthetic precursor of S‐TeA based on the structure analysis and early studies. B) HPLC‐MS chromatograms in positive ion mode of fermentation broth extracts of *A. alternata* cultured for 6 days without feeding AMHA (−AMHA) (above) and with the addition of AMHA on the second day after *A. alternata* inoculation (+AMHA) (below). C) Content of S‐TeA in −AMHA and +AMHA broths used for chromatography. Data shown are mean values ± SD of three independent biological replicates. Significant differences between −AMHA and +AMHA were determined according to Student's *t*‐test (two‐tailed). D) The known biosynthesis pathway of TeA in *M. oryzae* and the predicted biosynthesis pathway of S‐TeA based on structure analysis and early studies. E‐G) The content of S‐TeA, AMHA and N‐AcAc‐AMHA in *A. alternata* cultures after 6 days of growth, with threonine supplementation occurring on the second day following *A. alternata* inoculation, respectively. H) Analysis of the linear relationship between the content of AMHA or N‐AcAc‐AMHA and S‐TeA. Data are mean values ± SD of three independent biological replicates.

Fungal secondary metabolites such as polyketides and non‐ribosomal peptides are typically synthesized by PKSs and NRPSs. PKSs catalyze the condensation reactions of acyl‐CoA building blocks, while NRPSs unique incorporate amino acids into diverse bioactive non‐ribosomal peptides.^[^ ^41^, ^42^ ^]^ TeA is considered a hybrid molecule, composed of isoleucine and two acetate units, synthesized by a fungal PKS‐NRPS enzyme.^[^ ^12^, ^43^ ^]^ Feeding *A. tenuis* with valine or leucine results in the production of the respective isopropyl or isobutyl analogues of tenuazonic acid (**Figure** **4**A).^[^ ^17^, ^24^ ^]^ Therefore, it is hypothesized that the precursor of S‐TeA is 2‐amino‐3‐methylhexanoic acid (AMHA), also known as *β*‐methylnorleucine, a naturally occurring endogenous *α*‐amino acid.^[^ ^44^ ^]^

To validate AMHA as the immediate precursor of S‐TeA in the biosynthetic pathway, 20 mg L^−1^ AMHA was added to the fermentation broth of *A. alternata* to monitor S‐TeA production. Extracts from both the control (without AMHA, −AMHA) and the supplemented (with AMHA, +AMHA) fermentation broths had peaks corresponding to S‐TeA (*m*/*z* 212.1287, [M+H]^+^) with a retention time of 5.66 min after 6 days of culturing. The S‐TeA concentration in the +AMHA broth reached 6.2 mg L^−1^, a fourfold increase over the −AMHA control (Figure 4B,C), indicating that AMHA is indeed the immediate precursor of S‐TeA in *A. alternata*.

Recently, TeA synthetase 1 (TAS1, EC 6.3.2.50) was identified as the fungal NRPS‐PKS hybrid enzyme responsible for synthesizing TeA from *L*‐isoleucine (Ile) and acetoacetyl‐coenzyme A (AcAc‐CoA) via N‐AcAc‐Ile in *M*. *oryzae* and *A. alternata* (Figure 4D).^[^ ^45^, ^46^ ^]^ Structurally, S‐TeA, a *β*‐methylnorleucine‐derived 3‐acyltetramic acid, contains a sec‐pentyl alkyl group with five carbon atoms at 5‐position, contrasting with the sec‐butyl alkyl group with four carbon atoms in TeA. This structural similarity, along with the understanding of TeA biosynthesis provided a basis for investigating the biosynthetic pathway of S‐TeA.

Like isoleucine, AMHA is also a branched‐chain amino acid (BCAA) with an additional methyl group at the *δ*‐position. In fungi, threonine serves as the precursor for isoleucine in the BCAA biosynthetic pathway, occurring in the mitochondria.^[^ ^47^, ^48^, ^49^ ^]^ This raises the question of whether threonine also acts as the precursor for AMHA synthesis through the BCAA pathway (Figure 4D). To test this hypothesis, four amino acids, threonine, aspartic acid, arginine and isoleucine, which are curial precursors for BCAAs and other secondary metabolites as well as key feedback regulators of the BCAA biosynthetic pathway, were added to cultures of *A. alternata* to monitor the accumulation of AMHA, N‐AcAc‐AMHA, and S‐TeA. Of the four, only threonine caused a clear concentration‐dependent increase in AMHA, N‐AcAc‐AMHA, and S‐TeA levels in *A. alternata* (Figure 4E‐G, Figure S6A–C, Supporting Information). Increasing threonine concentrations from 5 to 40 mg L^−1^, resulted in S‐TeA increments ranging from 5% to 64% relative to the control (Figure 4G). Furthermore, a strong positive correlation between S‐TeA levels and the concentrations of AMHA or N‐AcAc‐AMHA confirms their crucial roles as intermediates in the S‐TeA biosynthetic pathway (Figure 4H).

Interestingly, a similar concentration‐dependent relationship was observed between threonine addition and the production of isoleucine, N‐AcAc‐isoleucine, and TeA (Figure S6D, Supporting Information). These results strongly suggest that threonine serves as a common biosynthetic precursor for both TeA and S‐TeA in *A. alternata*.

### 2.4 Discovery of Core Genes in the S‐TeA Biosynthetic Pathway in *A. alternata* Using Transcriptomics

To fully elucidate the biosynthetic pathway of S‐TeA in *A. alternata*, a transcriptomics analysis was conducted to profile gene expression following the addition of threonine to the culture. Differentially expressed genes (DEGs) were visualized using volcano plots, highlighting significant changes between treated and untreated groups (Figure S7A, Table S2, Supporting Information). Genes were categorized into ten clusters. Cluster 3 contained 1505 genes that were downregulated when threonine was added (+Thr) compared to the control without threonine (−Thr), whereas Cluster 7 contained 753 genes that were upregulated under the same conditions (Figure S7B, Table S3‐1, Supporting Information). A Gene Ontology (GO) enrichment was focused on biological processes for these clusters. In Cluster 3, the most significant enrichment of GO terms related to gene expression, RNA metabolic processes, and macromolecule modification (Table S3‐2, Supporting Information). Cluster 7 showed significant enrichment in biosynthetic and metabolic processes, including oxidation‐reduction, BCAA biosynthesis, small molecule biosynthesis, and *α*‐amino acid biosynthesis. (Table S3‐3, Supporting Information). These findings align with our hypothesis that the S‐TeA biosynthetic pathway in *A. alternata* involves into two stages: the formation of the key intermediate AMHA from threonine followed by the conversion of AMHA to S‐TeA.

**Figure 5:**
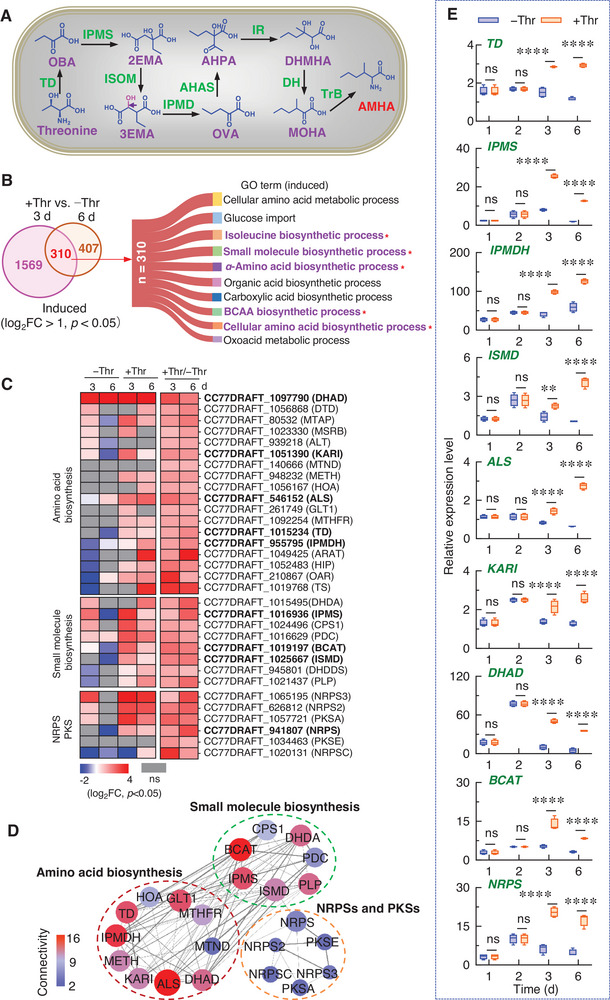
The key biosynthetic genes of S‐TeA produced in *A. alternata*. A) The predicted biosynthesis of AMHA in *E. coli* strain JM109. B) Venn diagram of unique and common up‐regulated at least twofold DEGs in the +Thr/−Thr group at 3 and 6 days (left). The Gene Ontology (GO) enrichment analysis of 310 common up‐regulated DEGs in the +Thr/−Thr group at 3 and 6 days (right). C) Heat map of differentially expressed genes enriched in amino acid biosynthesis, small molecule biosynthesis or non‐ribosomal peptide synthetases (NRPS) and polyketide synthase (PKS) in the groups of −Thr 3 d/ −Thr 1 d, −Thr 6 d/ −Thr 1 d, +Thr 3 d/ −Thr 1 d, +Thr 6 d/ −Thr 1 d, +Thr 3 d/ −Thr 3d and +Thr 6 d/ −Thr 6 d. D) The Protein‐Protein Interaction networks (PPI) of S‐TeA biosynthesis pathway. The circles are different proteins, the colors of different gradients and the size of the circles reflect the connectivity degree of corresponding proteins. The width of solid lines referred the closeness centrality degree of corresponding proteins. The brighter the color, larger the shape and the wider the line indicate the existence of more protein interactions. E) The expression levels of nine key S‐TeA biosynthetic genes of *A. alternata* determined using quantitative RT‐qPCR (normalized to *ACTIN*) in –Thr or +Thr groups cultured for 1, 2, 3 and 6 days, respectively. Data shown are mean values ± SD of four independent biological replicates. Asterisks indicate significant differences compared to −Thr at each time point by Student's *t*‐test (^*^ *p* < 0.05, ^**^ *p* < 0.01, ^***^ *p* < 0.001; ^****^ *p* < 0.0001). OBA, 2‐oxobutanoic acid; 2EMA, 2‐ethylmalic acid; 3EMA, 3‐ethylmalic acid; OVA, 2‐oxovaleric acid; AHPA, 2‐acetyl‐2‐hydroxypentanoic acid; DHMHA, 2,3‐dihydroxy‐3‐methylhexanoic acid; MOHA, 3‐methyl‐2‐oxohexanoic acid; AMHA, 2‐amino‐3‐methylhexanoic acid; TD, threonine deaminase; IPMS, 2‐isopropylmalate synthase; ISOM, *α*‐isopropylmalate isomerase; IPMD, *β*‐isopropylmalate dehydrogenase; AHAS, acetohydroxy acid synthase; IR, acetohydroxy acid isomeroreductase; DH, dihydroxy acid dehydratase; TrB, transaminase B; IPMDH, 3‐isopropylmalate dehydratase; ISMD, isopropylmalate dehydrogenase; ALS, acetolactate synthase; KARI, ketol‐acid reductoisomerase; DHAD, dihydroxy acid dehydratase; BCAT, branched‐chain amino acid aminotransferase.

Previous study predicted eight enzymes involved in AMHA synthesis from *L*‐threonine in the engineered *Escherichia coli* (**Figure** **5**A).^[^ ^50^ ^]^ These enzymes, associated with the isoleucine and leucine biosynthetic pathways, are threonine deaminase (TD, EC 4.3.1.19), acetohydroxy acid synthase (AHAS, EC 4.1.3.18), acetohydroxy acid isomeroreductase (IR, EC 1.1.1.86), dihydroxy acid dehydratase (DH, EC 4.2.1.9), and transaminase B (TrB, EC 2.6.1.42), encoded by the *Ilv1*, *Ilv2*, *Ilv5*, *Ilv3* and *Bat1* genes, respectively, as well as 2‐isopropylmalate synthase (IPMS, EC 4.1.3.12), *α*‐isopropylmalate isomerase (ISOM, EC 4.2.1.33) and *β*‐isopropylmalate dehydrogenase (IPMD, EC 1.1.1.85), encoded by the *LeuA*, *LeuCD* and *LeuB* genes, respectively.^[^ ^51^, ^52^ ^]^ This pathway suggested that AMHA synthesis in *A. alternata* could be similar to the pathway in engineered *E. coli* strain JM109. Analysis of the up‐regulated DEGs in the +Thr versus −Thr comparisons revealed 310 DEGs consistently upregulated at both 3 days and 6 days post treatment (Figure 5B, Table S4, Supporting Information). GO analysis of these genes showed significant enrichment for processes related to isoleucine biosynthesis, small molecule biosynthesis, *α*‐amino acids, BCAA and cellular amino acids.

Thirty‐two genes were selected for further study based on their roles in amino acid biosynthesis, small molecule biosynthesis and NRPS and PKS pathways (Figure 5C, Table S5, Supporting Information). Of the enzymes involved in AMHA synthesis in *E. coli* (Figure 5A), homologous genes were identified in *A. alternata*: TD and IPMS are encoded by *CC77DRAFT_1 015 234* and *CC77DRAFT_1 016 936*, respectively. Other enzymes in *A. alternata* include 3‐isopropylmalate dehydratase (IPMDH), isopropylmalate dehydrogenase (ISMD), ketol‐acid reductoisomerase (KARI), dihydroxy acid dehydratase (DHAD), and branched‐chain amino acid aminotransferase (BCAT) encoded by *CC77DRAFT_955* *795*, *CC77DRAFT_1 025 667*, *CC77DRAFT_1 051 390*, *CC77DRAFT_1 097 790* and *CC77DRAFT_1 019 197*, respectively. The gene encoding acetolactate synthase (ALS, EC 2.2.1.6) in *A. alternata*, *CC77DRAFT_546 152*, exhibited similar activity to AHAS in *E. coil*.^[^ ^50^, ^53^ ^]^ The log_2_ fold change (log_2_FC) values for genes *TD*, *IPMS*, *IPMDH*, *ISMD*, *ALS*, *KARI*, *DHAD* and *BCAT* were 1.36, 1.07, 1.44, 1.96, 2.25, 1.75, 2.74, and 2.27 in the group of +Thr/−Thr at 3 d, while their corresponding values in +Thr/−Thr groups at 6 days were 1.85, 2.53, 1.86, 2.09, 1.74, 1.61, 2.01, and 2.00, confirming the role of threonine in activating the biosynthetic pathway (Figure 5C, Table S5, Supporting Information).

Among the *NRPS* and *PKS* related genes, *CC77DRAFT_941 807* (*Aa*TAS1) encodes a hybrid NRPS/PKS enzyme with domains for adenylation (A), condensation (C), peptidyl carrier protein (PCP), and ketosynthase (KS).^[^ ^46^ ^]^ This gene shares 60.3% homology with *TAS1* in *M. oryzae* and has been linked to TeA production in *A. alternata*.^[^ ^45^, ^46^ ^]^ Expression of *CC77DRAFT_941 807* was significantly upregulated in the +Thr groups, suggesting a role in both TeA and S‐TeA biosynthesis due to structural similarities (Figure 5C, Table S5, Supporting Information).

Protein‐Protein Interaction (PPI) network analysis, of the 32 selected enzymes (Figure 5C) identified 23 forming a network, divided into three main groups: amino acid biosynthesis (TD, DHAD, IPMDH, KARI, ALS), small molecule biosynthesis (IPMS, ISMD, BCAT), and NRPS/PKS pathways (NRPS). The high connectivity of these genes supports their central role in S‐TeA synthesis (Figure 5D, Table S6, Supporting Information). The quantitative real‐time PCR (RT‐qPCR) results confirmed significantly higher expression of these genes in the +Thr group, consistent with the transcriptomics data (Figure 5E).

### 2.5 Metabolites in the S‐TeA Biosynthetic Pathway: Untargeted Metabolomics Analysis

**Figure 6:**
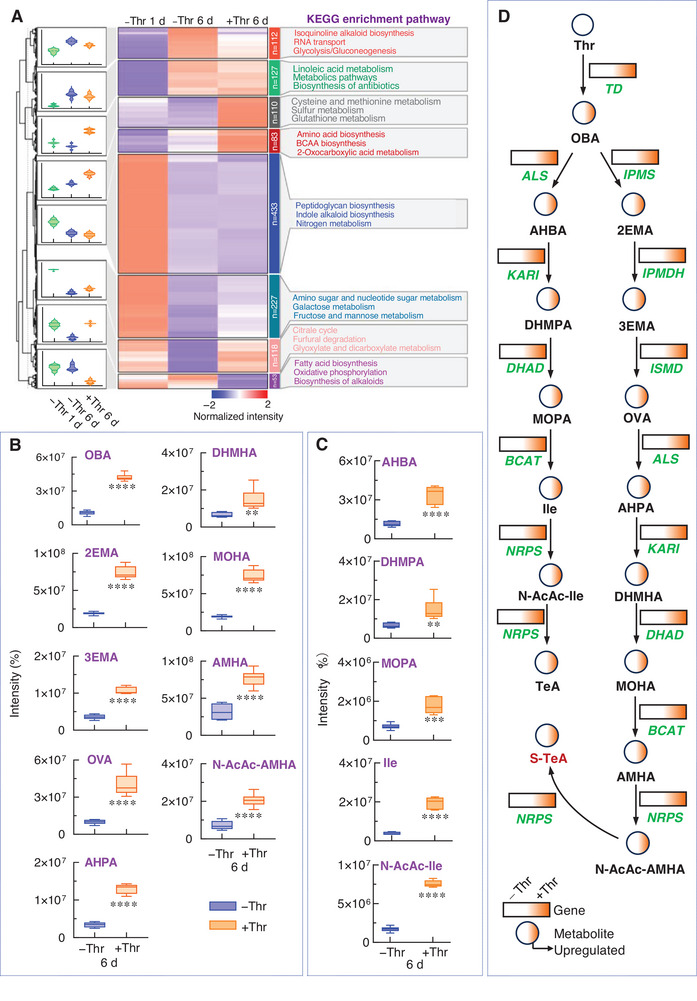
The key metabolites of the S‐TeA biosynthetic process on the basis of untargeted metabolomics. A) Multigroup heat map with normalized metabolites intensities and chart of Kyoto Encyclopedia of Genes and Genomes (KEGG) enrichment analysis of metabolites. B) and C) The key metabolites intensities of S‐TeA and TeA biosynthetic pathway of +Thr 6d and −Thr 6d, respectively. Significant differences (^*^ *p* < 0.05, ^**^ *p* < 0.01, ^***^ *p* < 0.001, ^****^ *p* < 0.0001) between +Thr 6 d and −Thr 6 d were determined according to Student's *t*‐test (two‐tailed) D) Biosynthetic pathways of TeA and S‐TeA based on the analysis of RNA‐seq and untargeted metabolomics. Key genes and metabolites were up‐regulated of *A. alternata* in the +Thr group compared with the −Thr group resulting in the accumulation of TeA and S‐TeA. Thr, threonine; OBA, 2‐oxobutanoic acid; Ac‐CoA, acetyl‐coenzyme A; 2EMA, 2‐ethylmalic acid; 3EMA, 3‐ethylmalic acid; OVA, 2‐oxovaleric acid; AHPA, 2‐acetyl‐2‐hydroxypentanoic acid; DHMHA, 2,3‐dihydroxy‐3‐methylhexanoic acid; MOHA, 3‐methyl‐2‐oxohexanoic acid; AMHA, 2‐amino‐3‐methylhexanoic acid; AHBA, 2‐acetyl‐2‐hydroxybutyric acid; DHMPA, 2,3‐dihydroxy‐3‐methylpentanoic acid; MOPA, 3‐methyl‐2‐oxopentanoic acid; Ile, isoleucine; TD, threonine deaminase; IPMS, 2‐isopropylmalate synthase; IPMDH, 3‐isopropylmalate dehydratase; ISMD, isopropylmalate dehydrogenase; ALS, acetolactate synthase; KARI, ketol‐acid reductoisomerase; DHAD, dihydroxy acid dehydratase; BCAT, branched‐chain amino acid aminotransferase; NRPS, non‐ribosomal peptide synthetases.

Previous studies suggested that threonine, rather than isoleucine is precursor of AMHA due to specific catalytic properties in the BCAAs biosynthesis pathway.^[^ ^50^ ^]^ To clarify the biosynthetic pathway, an untargeted metabolomic analysis was performed, identifying 1263 metabolites in *A. alternata* in both −Thr and +Thr conditions. Metabolites were grouped into eight clusters based on intensity patterns (Table S7, Supporting Information). Kyoto Encyclopedia of Genes and Genomes (KEGG) analysis highlighted that metabolites in Clusters 3 and 4 were significantly upregulated in the +Thr condition. In Cluster 3, 110 enriched metabolites corresponded to cysteine and methionine, sulfur, and glutathione metabolism, whereas in Cluster 4, 83 enriched metabolites were associated with amino acid and BCAA biosynthesis, and 2‐oxocarboxylic acid metabolism (**Figure** **6**A, Table S7, Supporting Information). These results matched the transcriptomic data (Figure 5B), indicating that upregulated genes expression promoted the production of key metabolites.

For tetramic acid derivatives like TeA, erythroskyrine, malonomicin, streptolydigin and aflastatin A, substitution at the C‐5 and C‐3 positions derive from amino acid and acyl moieties, respectively.^[^ ^7^, ^54^ ^]^ Analysis of differential metabolites in Cluster 3 and 4 revealed that key metabolites in the +Thr condition, including 2‐oxobutanoic acid (OBA), 2‐ethylmalic acid (2EMA), 3‐ethylmalic acid (3EMA), 2‐oxovaleric acid (OVA), 2‐acetyl‐2‐hydroxypentanoic acid (AHPA), 2,3‐dihydroxy‐3‐methylhexanoic acid (DHMHA), 3‐methyl‐2‐oxohexanoic acid (MOHA), AMHA and N‐AcAc‐AMHA, were significantly upregulated (Figure 6B, Table S7, Supporting Information). The increased abundance of N‐AcAc‐AMHA suggested that the C‐A‐PCP domain of NRPS catalyzes the combination of AMHA with AcAc‐CoA to produce N‐AcAc‐AMHA, while the KS domain recognized the N‐AcAc‐AMHA hybrid for cyclization, leading to the formation of S‐TeA. Further investigation confirmed that the intensities of metabolites OBA, 2‐acetyl‐2‐hydroxybutyric acid (AHBA), 2,3‐dihydroxy‐3‐methylpentanoic acid (DHMPA), 3‐methyl‐2‐oxopentanoic acid (MOPA), isoleucine (Ile) and N‐AcAc‐Ile associated with the TeA biosynthetic pathway, catalyzed by TD, AS, KARI, DHAD, BCAT and NRPS, were significantly upregulated in the +Thr group compared to the −Thr group at 6 days (Figure 6C, Table S7, Supporting Information). For Ile, the deammoniated product OBA was directly used in its synthesis. In contrast, the biosynthesis of AMHA, required three additional enzymatic steps involving OBA to extend the carbon skeleton, ultimately producing OVA. By integrating transcriptomics and untargeted metabolomics analyses, we identified and demonstrated the key genes and the enzyme‐catalyzed metabolic processes involved in the biosynthetic pathway of S‐TeA and TeA in *A. alternata*. The addition of threonine promoted the upregulation of these genes, leading to the accumulation of their corresponding metabolites (Figure 6D).

### 2.6 Definition of the Key Steps in the S‐TeA Biosynthetic Pathway Using Isotope‐Label Precursor Tracing

**Figure 7:**
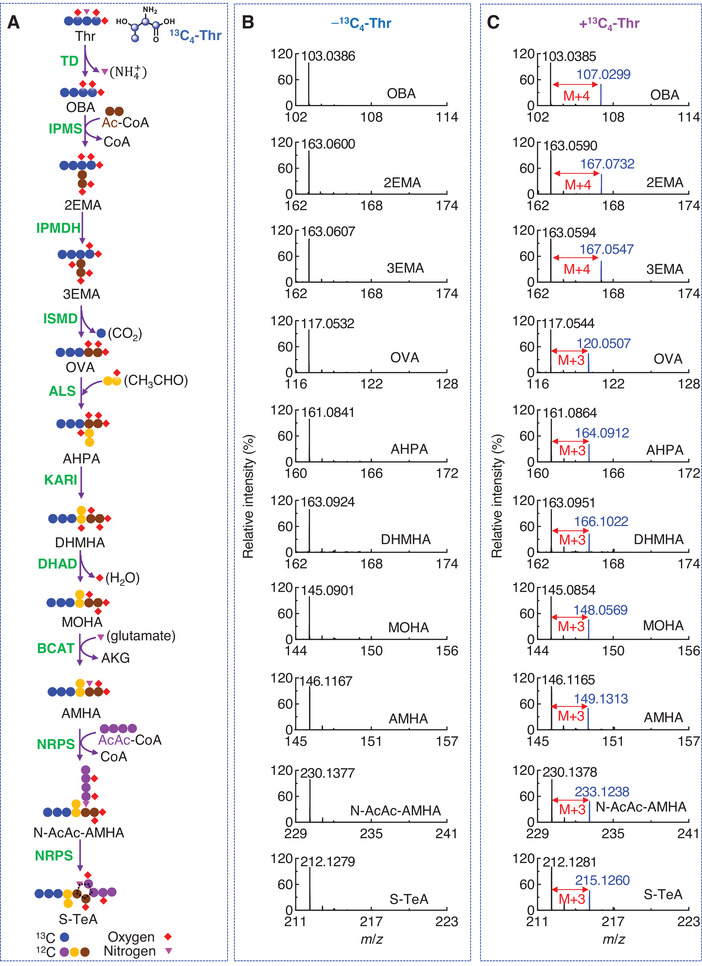
The key steps of the S‐TeA biosynthetic pathway using uniformly ^13^C‐labeled threonine (^13^C_4_‐Thr) in *A* ***. alternata***. A) The routes for biosynthesis of S‐TeA and its predicted labeling from ^13^C_4_‐Thr. B) Mass spectra of key metabolites synthesized from unlabeled threonine (−^13^C_4_‐Thr) in *A. alternata*. C) Mass spectra of key metabolites synthesized from ^13^C_4_‐Thr (+^13^C_4_‐Thr) in *A. alternata*. Unlabeled and newly synthesized compounds from ^13^C_4_‐Thr labeled represented by black and blue lines, respectively. The response of the most abundant peak in each graph was set to 100%. Thr, threonine; OBA, 2‐oxobutanoic acid; Ac‐CoA, acetyl‐coenzyme A; 2EMA, 2‐ethylmalic acid; 3EMA, 3‐ethylmalic acid; OVA, 2‐oxovaleric acid; AHPA, 2‐acetyl‐2‐hydroxypentanoic acid; DHMHA, 2,3‐dihydroxy‐3‐methylhexanoic acid; MOHA, AKG, *α*‐ketoglutaric acid; 3‐methyl‐2‐oxohexanoic acid; AMHA, 2‐amino‐3‐methylhexanoic acid; TD, threonine deaminase; IPMS, 2‐isopropylmalate synthase; IPMDH, 3‐isopropylmalate dehydratase; ISMD, isopropylmalate dehydrogenase; ALS, acetolactate synthase; KARI, ketol‐acid reductoisomerase; DHAD, dihydroxy acid dehydratase; BCAT, branched‐chain amino acid aminotransferase; NRPS, non‐ribosomal peptide synthetases.

To further elucidate the S‐TeA biosynthetic pathway and its differences from TeA in *A. alternata*, stable isotope feeding experiments were conducted to track the label‐patterns of key metabolites involved in the TeA and S‐TeA biosynthetic process. When uniformly labeled ^13^C‐threonine (^13^C_4_‐Thr) was administered, an additional peak corresponding to [M+H+4]^+^ was detected in the MS spectra. This resulted in the enrichment of ^13^C_4_‐OBA (*m/z* 107.0299), ^13^C_4_‐AHBA (*m/z* 151.0784), ^13^C_4_‐DHMPA (*m/z* 153.0903), ^13^C_4_‐MOPA (*m/z* 107.0299), ^13^C_4_‐Ile (*m/z*135.0407), ^13^C_4_‐N‐AcAc‐Ile (*m/z* 220.0949) and ^13^C_4_‐TeA (*m/z* 202.1148) compared to the non‐labeled control (−^13^C_4_‐Thr) (Figures S8A–C and S9, Supporting Information). These results confirmed that four ^13^C‐atoms from threonine were incorporated into TeA, validating previously established biosynthetic pathway (Figure S8A, Supporting Information).^[^ ^45^ ^]^ For the S‐TeA biosynthetic pathway, additional ^13^C_4_ signals ([M+H+4]^+^) were observed for OBA (*m/z* 107.0299), 2EMA (*m/z* 167.0732) and 3EMA (*m/z* 167.0547) in the +^13^C_4_‐Thr group. In contrast, ^13^C_3_ signals ([M+H+3]^+^) were detected for OVA (*m/z* 120.0507), AHPA (*m/z* 164.0912), DHMHA (*m/z* 166.1022), MOHA (*m/z* 148.0854), AMHA (*m/z* 149.1313), N‐AcAc‐AMHA (*m/z* 233.1238), and S‐TeA (*m/z* 215.1260) (**Figure** **7**A–C, Figure S10, Supporting Information). Our results, alongside previous studies (Figure 6D) suggest that AMHA synthesis involves enzymes from the Ile and Leu biosynthesis pathways.^[^ ^52^ ^]^ In Leu biosynthesis, the formation of the isobutyl side chain requires a transfer of the isopropyl group from C2 to C3 of 2‐isopropylmalate, followed by a decarboxylation step catalyzed sequentially by IPMDH and ISMD.^[^ ^55^, ^56^, ^57^ ^]^ A similar mechanism occurs in homoleucine biosynthesis, where IPMDH and ISMD catalyze the conversion of 2‐isobutylmalate to 4‐methyl‐2‐oxopentanoate.^[^ ^42^ ^]^

In our study, the stable isotope signal shifted from ^13^C_4_ to ^13^C_3_ during OVA formation (Figure 7C), indicating a decarboxylation step likely catalyzed by ISMD.^[^ ^58^ ^]^ This suggest that the ethyl group of 2EMA migrates to the C3 position, forming 3EMA. Subsequently, ISMD catalyzes the decarboxylation of 3EMA to OVA (Figure 7A), retaining only three carbons from threonine in the final metabolites, including S‐TeA.

### 2.7 IPMS, IPMDH, and ISMD Are Key Enzymes for Extending the Carbon Skeleton of S‐TeA

Based on previous analyses, nine key genes and corresponding metabolites involved in the S‐TeA biosynthesis in *A. alternata* were identified. Among these, three specific genes, *IPMS*, *IPMDH* and *ISMD*, were unique to the S‐TeA biosynthetic pathway and absent from the TeA pathway. These findings suggest that the distinct differences between the biosynthesis of S‐TeA and TeA are due to reactions catalyzed by these three enzymes.

**Figure 8:**
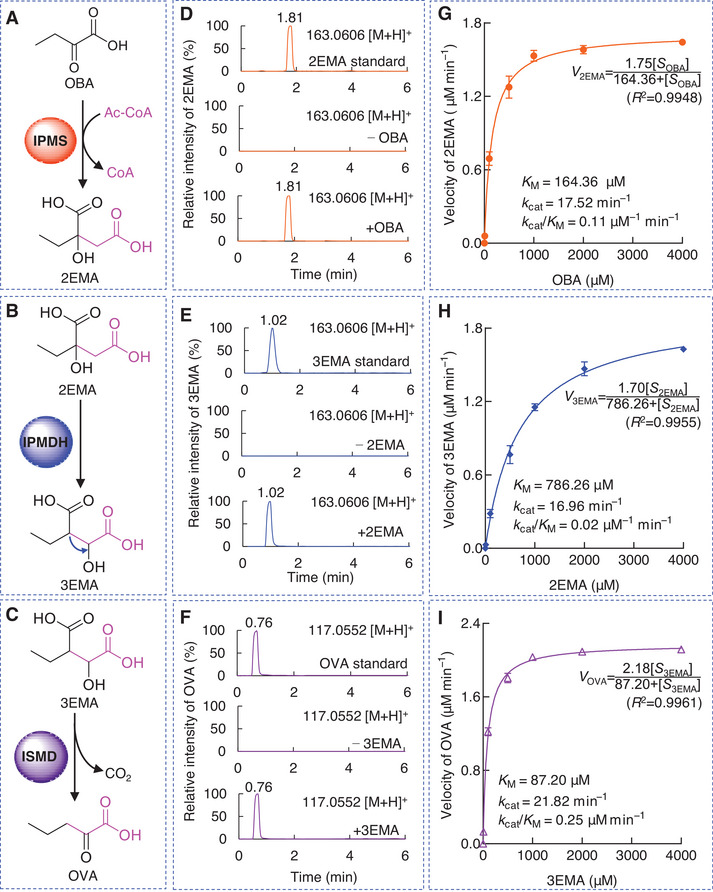
Biochemical analysis of three vital enzymes, IPMS, IPMDH and ISMD with corresponding respective substrates, OBA, 2EMA and 3EMA. Schematic view of enzymatic reaction from A) OBA to 2EMA catalyzed by IPMS, B) 2EMA to 3EMA catalyzed by IPMDH and C) 3EMA to OVA catalyzed by ISMD. D) UPLC‐MS chromatograms in positive mode of 2EMA standard (above), and 2EMA produced through in vitro biochemical reactions catalyzed by IPMS with 0 µM (−OBA, middle) or 1000 µM OBA (+OBA, below) as substrates. E) UPLC‐MS chromatograms in positive mode of 3EMA standard (above), and 3EMA produced through in vitro biochemical reactions catalyzed by IPMDH with 0 µM (−2EMA, middle) or 1000 µM 2EMA (+2EMA, below) as substrate. F) UPLC‐MS chromatograms in positive mode of OVA standard (above), and OVA produced through in vitro biochemical reactions catalyzed by ISMD with 0 µM (−3EMA, middle) or 1000 µM 3EMA (+3EMA, below) as substrates. The Michaelis‐Menten model and kinetic parameters of G) IPMS toward substrate OBA by monitoring 2EMA accumulation, H) IPMDH toward substrate 2EMA by monitoring 3EMA accumulation, and I) ISMD toward substrate 3EMA by monitoring OVA accumulation. Data shown are mean values ± SD of three independent biological replicates. OBA, 2‐oxobutanoic acid; Ac‐CoA, acetyl‐coenzyme A; 2EMA, 2‐ethylmalic acid; 3EMA, 3‐ethylmalic acid; OVA, 2‐oxovaleric acid; IPMS, 2‐isopropylmalate synthase; IPMDH, 3‐isopropylmalate dehydratase; ISMD, isopropylmalate dehydrogenase.

We examined these three enzymatic steps in *vitro* (**Figure** **8**A–C). As expected, when OBA served as a substrate for IPMS, a new peak appeared, displaying an increase of 60 *m/z* ([M+H]^+^) over OBA at a retention time of 1.81 min in the positive mode, corresponding to the 2EMA standard (*m/z* 163.0606). This increase was attributed to a condensation reaction between OBA and Ac‐CoA (Figure 8D). Additionally, IPMDH exhibited isomerization activity; as a new peak appeared with the same parent ion as 2EMA in the positive mode, but with a different retention time (1.02 min), corresponding to the 3EMA standard (Figure 8E). Furthermore, a decrease of 46 *m/z* ([M+H]^+^) was observed when 3EMA was processed in the reaction catalyzed by ISMD, indicating decarboxylation and conversion of 3EMA to OVA (Figure 8F).

To gain a deeper insight into the catalytic functions of IPMS, IPMDH and ISMD, their kinetic properties were assessed by quantifying product accumulation (Figure 8G–I). The kinetic profiles of the three substrates followed a classic Michaelis–Menten behavior. The Michaelis constant (*K*_M_) for IPMS in the condensation reaction with OBA was 164.36 µM, significantly lower than IPMDH's *K*_M_ value of 786.26 µM in the isomerization reaction with 2EMA, but higher than ISMD's *K*_M_ value of 87.20 µM in the decarboxylation of 3EMA. Additionally, turnover constants (*k*_cat_) were calculated for IPMS, IPMDH and ISMD with their respective substrates, yielding catalytic efficiency values (*k*_cat_/*K*_M_) of 0.11 µM^−1^ min^−1^, 0.02 µM min^−1^ and 0.25 µM^−1^ min^−1^, respectively. A lower *K*_M_ value indicates a better substrate binding, while a higher *k*_cat_/*K*_M_ value indicates greater catalytic efficiency.^[^ ^59^ ^]^ Notably, IPMDH was the key rate‐limiting enzyme in AMHA biosynthesis as it exhibited lower affinity for 2EMA compared to the affinities of IPMS and ISDM for their respective substrates. Condensation, isomerization, and decarboxylation, catalyzed by unique IPMS, IPMDH, and ISMD, respectively, extend the carbon chain of OBA to form OVA, which is a critical key step that differentiates the S‐TeA pathway from TeA biosynthesis. This may explain why the content of the unique non‐protein amino acid AMHA (≈4 mg kg^−1^ FW) is significantly lower than that of the conventional protein amino acid Ile (≈94 mg kg^−1^ FW) in *A. alternata* culture (Figure 4E, Figure S6D, Supporting Information). Evaluation of the NRPS (*Aa*TAS1) substrate specificity for the immediate precursor of TeA (Ile) and S‐TeA (AMHA) revealed that Ile and AMHA exhibited similar binding affinities to the A domain of *Aa*TAS1 (Figure S11 and Table S8, Supporting Information). Thus, the primary reason for the markedly lower production of S‐TeA compared to TeA is the limited availability of AMHA relative to Ile in the culture, rather than the substrate preference of *Aa*TAS1. Future studies should focus on modifying the three key enzymes, particularly IPMDH, to improve substrate affinity or creating a novel synthetic pathway for S‐TeA in engineered strains, aiming to enhance S‐TeA production efficiency.

**Figure 9:**
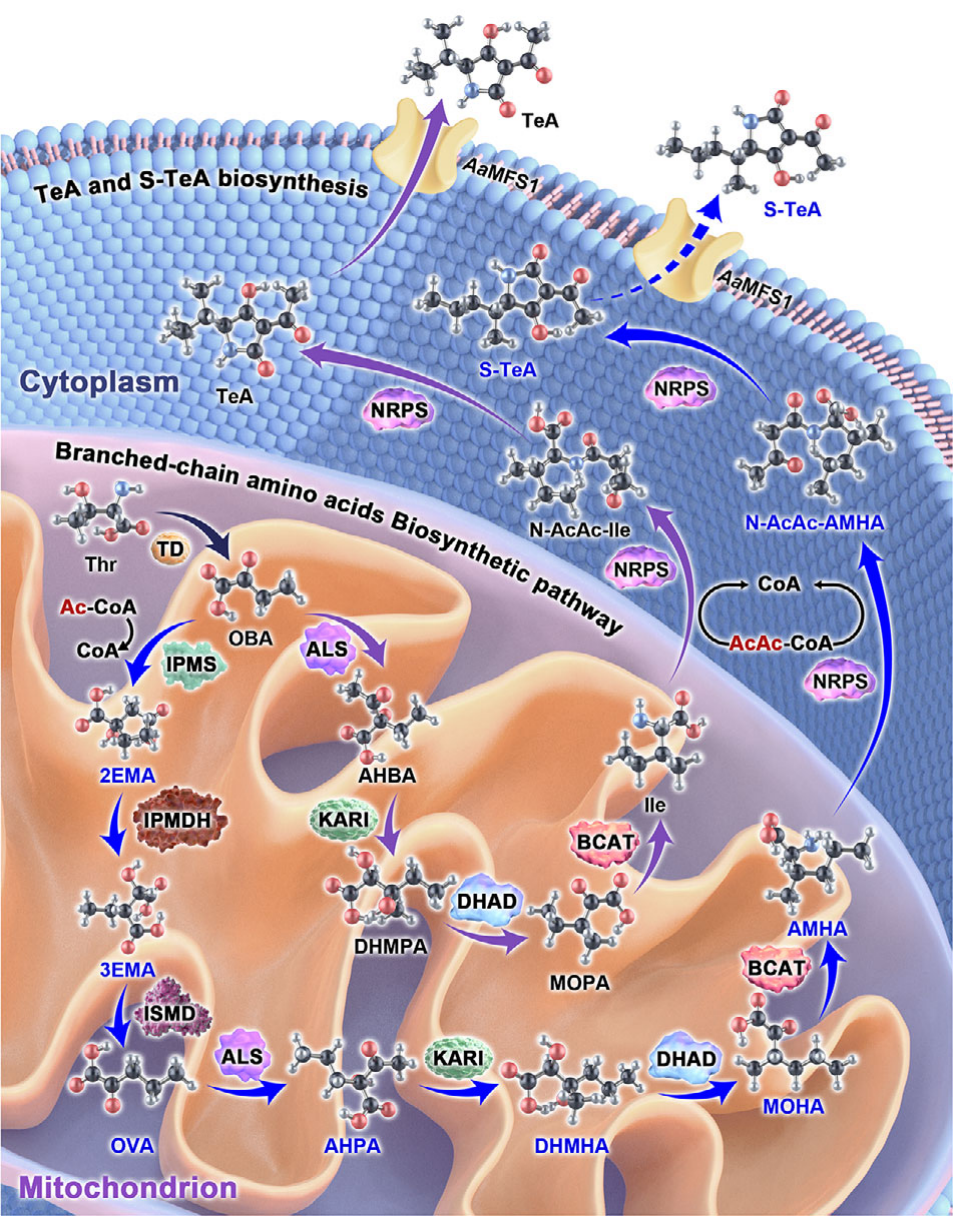
A proposed model for the biosynthetic pathway of S‐TeA in *A. alternata*. The precursor threonine is initially converted to the AMHA intermediate by reactions catalyzed by eight enzymes belonging to branched‐chain amino acid biosynthetic pathway in mitochondrion. AMHA is then transported to the cytosol, where acetyl groups are incorporated before being cyclized by NRPS. Finally, S‐TeA is released outside the cell. For comparison, the biosynthetic pathway of TeA is included. Carbon atoms are shown in black, nitrogen in blue, oxygen in red and hydrogen in gray. Each enzyme is displayed as molecular surfaces that can be distinguished by shape and color. The common enzyme reaction to the S‐TeA and TeA biosynthetic pathway is marked by an indigo arrow. The unique enzyme reactions of S‐TeA and TeA biosynthesis are marked as blue and purple arrows, respectively. Thr, threonine; OBA, 2‐oxobutanoic acid; Ac‐CoA, acetyl‐coenzyme A; 2EMA, 2‐ethylmalic acid; 3EMA, 3‐ethylmalic acid; OVA, 2‐oxovaleric acid; AHPA, 2‐acetyl‐2‐hydroxypentanoic acid; DHMHA, 2,3‐dihydroxy‐3‐methylhexanoic acid; MOHA, 3‐methyl‐2‐oxohexanoic acid; AMHA, 2‐amino‐3‐methylhexanoic acid; AHBA, 2‐acetyl‐2‐hydroxybutyric acid; DHMPA, 2,3‐dihydroxy‐3‐methylpentanoic acid; MOPA, 3‐methyl‐2‐oxopentanoic acid; Ile, isoleucine; TD, threonine deaminase; IPMS, 2‐isopropylmalate synthase; IPMDH, 3‐isopropylmalate dehydratase; ISMD, isopropylmalate dehydrogenase; ALS, acetolactate synthase; KARI, ketol‐acid reductoisomerase; DHAD, dihydroxy acid dehydratase; BCAT, branched‐chain amino acid aminotransferase; NRPS, non‐ribosomal peptide synthetases; *Aa*MFS1, major facilitator superfamily protein 1 in *A. alternata*.

We propose the biosynthetic pathway of S‐TeA in *A. alternata* (**Figure** **9**). Initially, threonine undergoes deamination by TD to produce OBA, a precursor for both Ile and AMHA biosynthesis. To further extend the carbon chain, IPMS, IPMDH, and ISMD catalyze condensation, isomerization, and decarboxylation reactions to produce OVA. Unlike TeA, these additional enzyme‐mediated reactions extend the carbon side chain in S‐TeA. Subsequently, four reactions catalyzed by AS, KARI, DHAD, and BCAT convert OVA to AMHA, following the same pathway as in TeA biosynthesis. AMHA is then transferred from the mitochondrion to the cytosol, where it undergoes acetylation to form N‐AcAc‐AMHA, which is subsequently cyclized by NRPS to form S‐TeA.

The occurrence of natural product biosynthesis, metabolism and signaling required a number of transporters. Previous studies suggested that the gene *AaMFS1* (*CC77DRAFT_269 024*), which codes for a major facilitator superfamily (MFS) protein, might be responsible for the transmembrane transport of TeA in *A. alternata*.^[^ ^46^ ^]^ Given the structural similarities between S‐TeA and TeA, S‐TeA transmembrane transport may also be regulated by the same protein. Based on RT‐qPCR results, the expression level of the *AaMFS1* gene was significantly higher in the +Thr group compared to the −Thr group, indicating that *Aa*MFS1 may be involved in S‐TeA transport (Figure S12A, Supporting Information). To investigate this, the model of *Aa*MFS1 was constructed using AlphaFold3. Its domain structure analysis revealed an MFS superfamily domain spanning residues Val26 to Phe499 (Figure S12B, Supporting Information). Both TeA and S‐TeA could be modeled within *Aa*MFS1, analysis showed that both molecules were nestled within the cavity formed by five *α*‐helixes including residues from Leu21 to Thr41, residues from Tyr65 to Leu75, residues from Val88 to Leu101, residues from Ala115 to His133 and residues from Trp175 to Val196, with openings at the top and bottom (Figure S12C,D, Supporting Information). Residue Thr126 appeared to provide a critical hydrogen bond to the amino group of TeA or S‐TeA, and ≈90% of the amino acid residues involved in their binding pockets were identical (Figure S12E,F, Supporting Information), suggesting they played a significant role in regulating the gate of these openings for S‐TeA's transport across the plasma membrane. However, further molecular and genetic studies are needed to fully understand the role of *Aa*MFS1 in S‐TeA transmembrane transport.

These findings highlight that although threonine is a common precursor for both S‐TeA and TeA, their biosynthetic pathways are significantly different. The unique genes involved in S‐TeA biosynthesis and transport were essential and crucial for future biomanufacturing applications.

## 3 Conclusion

Our research has established that S‐TeA is a novel natural product synthesized by a variety of filamentous fungi. Chiral HPLC and ECD analyses confirmed the absolute configuration of Nat‐S‐TeA and its relationship to structural and bioactive properties. Additionally, we identified threonine as a precursor, along with nine key biosynthetic genes and the corresponding metabolites they produce for S‐TeA synthesis in *A. alternata*, confirming the biosynthetic mechanism. Despite sharing threonine as a common precursor and utilizing the transmembrane transporter *Aa*MFS1, the biosynthetic pathway of S‐TeA differs from that of TeA due to the involvement of three unique enzymes, IPMS, IPMDH, and ISMD. This study provides foundational biochemical information for designing efficient strategies for the industrial biomanufacturing of S‐TeA, facilitating its development as a commercial herbicide.

## 4 Experimental Section

### Chemicals

Standards of OBA (CAS No. 600‐18‐0), 2EMA (CAS No. 1944‐62‐3), 3EMA (CAS No. 16048‐76‐3), AHBA (CAS No.3142‐65‐2), DHMPA (CAS No. 562‐43‐6), MOPA (CAS No. 1460‐34‐0), and Ile (CAS No. 73‐32‐5) were obtained from Bide Pharmatech Co., Ltd. (Shanghai, China). AHPA (CAS No. 35178‐09‐7) and DHMDA (CAS No. 1822523‐95‐4) were purchased from Atomax Chemicals Co., Ltd. (Shenzhen, Guangdong, China). ^13^C‐labeled threonine (^13^C_4_‐Thr, CAS No: 55443‐53‐3) for isotope‐label precursor tracing experiments was obtained from Sigma‐Aldrich (Shanghai, China). OVA, MOHA, AMHA, N‐AcAc‐AMHA, N‐AcAc‐isoleucine, TeA and S‐TeA were synthesized in‐house and confirmed by proton NMR (^1^HNMR) and carbon NMR (^13^C NMR) spectrometry. Details of the synthetic methods and corresponding ^1^H NMR and ^13^C NMR data are available in the **Supplemental Methods**. Unless specified otherwise, additional chemicals were sourced from Sinopharm Chemical Reagent Co., Ltd. (Shanghai, China) or Aladdin Biochemical Technology Co., Ltd. (Shanghai, China).

### Fungal Strains

*A. alternata* (Fr.) Keissler wild‐type strain NEW001 was isolated from the invasive plant *A. adenophora*.^[^ ^60^ ^]^ *A. alternata* f. sp. *lycopersici* (No. Bio‐23742) was obtained from Beijing Biobw Co., Ltd. (Beijing, China). *A. brassicicola* (No. CGMCC 3.7804) was purchased from the China General Microbiological Culture Collection Center (CGMCC, Beijing, China). The *M. oryzae* Guy11 strain was provided by Prof. M. Zhou (College of Plant Protection, Nanjing Agricultural University, China).

### Plant Materials

*A. adenophora* was grown in a greenhouse for ≈6 months from rooted stem cuttings in a perlite‐vermiculite‐peat mixture (0.5:1:3, v/v) under a 12‐h photoperiod at 200 µmol (photons) m^−2^ s^−1^, 70% relative humidity, and 20–25 °C.

### Liquid Fermentation, Purification, and Characterization of S‐TeA

*A. alternata* NEW001 was cultured on potato dextrose agar (PDA) medium. Ten 5‐mm‐diameter agar discs taken from the growing edge of the culture were inoculated into 500 mL of Czapek liquid medium in 1000 mL flasks and incubated at 25 °C with shaking at 140 rpm for 7 days in darkness.^[^ ^17^ ^]^ Mycelia and fermentation broths were separated by centrifugation at 8000 × *g* for 10 min. The pH of the fermentation broth was adjusted to 2.0 using 2 M HCl, followed by triple extraction with ethyl acetate. Organic layers were dried, filtered, and concentrated under vacuum to yield crude extracts. Isolation and purification of S‐TeA was conducted a MPLC system (NGC Quest 10 Plus Chromatography System, Bio‐Rad Laboratories, Inc., Hercules, CA, USA) equipped with a SunFire Prep C_18_ column (10 × 250 mm, 10 µm, Waters, USA). Detection was performed at 256 nm with a mobile phase of 0.1% formic acid (*v*/*v*) in water and acetonitrile (40:60, *v*/*v*) at a flow rate of 2.0 mL min^−1^ at room temperature. Fractions corresponding to the retention time of 20 min were collected and freeze‐dried under vacuum for 24 h, yielding orange crystals of S‐TeA.

The crystals were analyzed using an Acquity UPLC system (Waters, USA) coupled to a Waters G2‐XS Q‐TOF mass spectrometer.^[^ ^44^ ^]^ UPLC separations were carried out with a binary solvent delivery system and an auto‐sampler equipped with a Waters Acquity BEH C18 column (2.1 mm *×* 100 mm, 1.7 µm particles). The flow rate was set to 0.4 mL min*^−^* ^1^, and 2 µL sample was injected for detection at 280 nm. The mobile phase consisted of two components: (solvent A) water containing 0.1% formic acid and 2 mM NH_4_Ac, and (solvent B) acetonitrile containing 0.1% formic acid and 2 mM NH_4_Ac. The gradient program was as follows: 5% solvent B was maintained for 1 min, increased linearly to 95% over 9 min, held at 95% for 2 min, and then reduced to 5% within 1 min and held at 5% for 1 min. Mass detection was performed using an electrospray ionization in positive ion mode, with a scan range of 50−1200 *m*/*z*. Leucine‐enkephalin (*m*/*z* 556.2771) was used as the lock mass for real‐time recalibration. The ionization parameters were optimized as follows: capillary voltage, 3.0 kV; cone voltage, 30 V; source temperature, 120 °C; and desolvation gas temperature, 400 °C. The collision energy was set to 20−40 eV. Data acquisition and processing were conducted using Masslynx 4.1 software (Waters, MA, USA). Extracted ion chromatograms (EICs) were generated from the total ion chromatogram (TIC) by extracting centroid spectra peaks with a width of 0.01 Da.

The IR spectra were recorded according to previous reference.^[^ ^61^ ^]^ on a NicoletTM 460 FT‐IR spectrophotometer (Thermo Fisher Scientific, USA), and analyzed with OMNIC software (Thermo Fisher Scientific, USA). Nuclear magnetic resonance (NMR) spectra were recorded on a JEOL ECX‐500 (500 MHz) spectrometer (Tokyo, Japan) using methanol‐*d*_4_ as solvent. Chemical shifts were referenced to the residual methanol‐*d*_4_ signals (^1^H, *δ* = 3.31 ppm, ^13^C, *δ* = 49.00 ppm). MestReNova 12.0 software (Mestrelab Research SL, Spain) was used for NMR data analysis.

### The Physical and Spectroscopic Data of S‐TeA

S‐TeA appeared as orange crystals; IR (KBr) *ν*_max_ 3667, 3200, 2956, 2920, 2860, 1713, 1661, 1617 cm^−1^. ^1^H NMR (Methanol‐*d*_4_, 500 MHz): *δ* 3.83 (1H, s, C *H* NH), 2.38 (3H, s, COC *H*_3_), 2.04–1.97 (1H, m, C *H* CH_3_), 2.05 (1H, s, C *H* CH3), 1.51–1.40 (2H, m, CH3CH2C*H*2), 1.42–1.37 (2H, m, CH_3_C *H*_2_CH_2_), 1.30–1.23 (2H, m, CH_3_CH_2_C *H*_2_), 0.91 (3H, t, *J* = 10 Hz, C *H*_3_CH), 0.72 (3H, d, *J* = 5.0 Hz, C *H*_3_CH_2_); ^13^C NMR (Methanol‐*d*_4_, 125 MHz): *δ* 197.81 (*C* OCH_3_), 187.33 (*C* OH), 175.28 (*C* ONH), 102.75 (*C* CO), 64.94 (*C* HNH), 35.76 (CH_3_ *C* H_2_CH_2_), 34.42 (CH_3_CH_2_ *C* H_2_), 20.06 (CO *C* H_3_), 13.10 (CH_3_ *C* H), 12.24 (*C* H_3_CH), 10.28 (*C* H_3_CH_2_); HR‐ESI‐MS *m/z* 212.1289 [M + H]^+^ (calcd for C_11_H_17_NO_3_, 212.1287).

### Test of S‐TeA in Different Fungal Strains

To quantitatively assess the yield of S‐TeA in *A. alternata*, *A*. *alternata* f. sp. *Lycopersici*, *A*. *brassicicola* and *M*. *oryzae*, crude extracts from 500 mL of Czapek liquid medium for each strain were collected and analyzed by UPLC‐MS. The extraction and detection of S‐TeA and TeA were performed using the method described above. Quantifications of S‐TeA and TeA were achieved using an external calibration curve. Standard solutions for S‐TeA and TeA were prepared in methanol at concentration ranges of 0.125–4 and 2.5–80 mg L^−1^, respectively, through gradient dilution.

### In Vitro Phytotoxicity and Photosynthesis Inhibiting Activity Assessment

The phytotoxicity of S‐TeA to *A. adenophora* was evaluated following the method described in the previous studies.^[^ ^14^ ^]^ Wound sites on *A. adenophora* leaves were treated with 20 µL of 0.1% methanol (control), Nat‐S‐TeA, Chem‐S‐TeA, or diastereomers (5*S*, 6*S*)‐S‐TeA, (5*S*, 6*R*)‐S‐TeA, (5*R*, 6*S*)‐S‐TeA or (5*R*, 6*R*)‐S‐TeA at concentrations of 50, 100, 200, and 400 µM for 48 h. Lesion diameters on the leaves were measured after 48 h.

For assessing photosynthesis‐inhibiting activity, 250 µL of thylakoid suspensions (200 µg Chl mL^−1^) were incubated in the dark for 30 min at 4 °C with the stereoisomers of S‐TeA at final concentrations of 0 (0.1% methanol), 25, 50, 100, 200, or 400 µM. Treated thylakoids, containing 40 µg chlorophyll, were then added to the reaction medium. The rate of oxygen evolution of PSII was measured referring to as previous study.^[^ ^62^ ^]^ All experiments were conducted in three independent biological replicates.

Chlorophyll *a* fluorescence rise kinetics were determined using a Plant Efficiency Analyzer (Hansatech Instruments Ltd., King's Lynn, UK) according to previous study.^[^ ^37^ ^]^ Leaf discs (7‐mm in diameter) from *A. adenophora* were immersed in solution of control, (5*S*, 6*S*)‐S‐TeA, (5*S*, 6*R*)‐S‐TeA, (5*R*, 6*S*)‐S‐TeA or (5*R*, 6*R*)‐S‐TeA at varying concentrations, and incubated for 6 h at room temperature in the dark. The raw data were analyzed using the Biolyzer 4HP V3.0 software.^[^ ^35^, ^37^ ^]^

### Configurational Analysis of Natural S‐TeA

Chiral HPLC analysis of Nat‐S‐TeA and Chem‐S‐TeA, as well as the chiral separation of Chem‐S‐TeA was performed on an Agilent Infinity 1260 HPLC instrument (Agilent Technologies Inc., CA, USA) using a Daicel CHIRALPAK IG‐3 column (250 × 4.6 mm, 3 µm) (Daicel Chemical Ltd., Tokyo, Japan). Detection was conducted at 256 nm, with a mobile phase consisting of a hexane/ethanol mixture (60:40, *v*/*v*) at a flow rate of 1.0 mL min^−1^.

ECD spectra of Nat‐S‐TeA and Chem‐S‐TeA were recorded on a JASCO J‐1500 CD Spectrophotometer (JASCO Co., Tokyo, Japan). Solutions of 100 µM Nat‐S‐TeA or Chem‐S‐TeA in methanol were prepared for ECD measurements. To determine their absolute configurations, theoretical ECD spectra of (5*S*, 6*S*)‐S‐TeA, (5*S*, 6*R*)‐S‐TeA, (5*R*, 6*S*)‐S‐TeA, and (5*R*, 6*R*)‐S‐TeA were generated using previously reported method.^[^ ^63^ ^]^ Conformational analysis was performed under the MMFF94 force field using the Molecular Operating Environment software (version 2016, Chemical Computing Group Inc., Montreal, QC, Canada), with an energy cutoff of 2.5 kcal mol^−1^ to ensure a broad distribution of conformers. Stable conformers were re‐optimized using density functional theory at the B3LYP/6‐31G (d) level in Gaussian 09 software (Gaussian Inc., Wallingford, CT, USA). Then, ECD calculations were conducted using time‐dependent density functional theory (TDDFT) at the B3LYP/6‐311+G (d, 2p) level with conductor‐like polarizable continuum (CPCM) solvation model in water. The ECD spectra were simulated using an overlapping Gaussian function, applying a half‐bandwidth at 1/e peak height (*σ* = 0.20) for all transitions. The final computational ECD spectra were derived by weighting the contributions of each conformer based on Boltzmann distribution.

### Modeling of S‐TeA in the Q_B_ Site

Discovery Studio (version 2019, BIOVIA, USA) was used to model S‐TeA in the Q_B_ site. The homology model of the D1 protein of *A. adenophora*, ligand preparation ((5*S*, 6*S*)‐S‐TeA, (5*S*, 6*R*)‐S‐TeA, (5*R*, 6*S*)‐S‐TeA or (5*R*, 6*R*)‐S‐TeA), binding site definition, and molecular docking methods were performed according to our previous study.^[^ ^14^ ^]^

### Feeding Experiments

Previous research suggested isoleucine was the biosynthetic precursor of TeA. The addition of leucine or valine to the fermentation broth of *A. tenuissima* has been shown to induce the synthesis of TeA derivatives with isobutyl or isopropyl chains at the 5‐position.^[^ ^7^ ^]^ Given the presence of an *α*‐amino acid structural unit corresponding to AMHA in S‐TeA, 20 mg L^−1^ AMHA was added to the fermentation broth of on the second day after *A. alternata* inoculation and incubated for an additional 4 days. Quantification of S‐TeA and TeA was performed using the method described above. Experiments were performed in three independent biological replicates.

To identify the biosynthetic precursor of S‐TeA, threonine, aspartic acid, arginine, and isoleucine were added to *A. alternata* cultures at final concentrations of 5, 10, 20, or 40 mg L^−1^ on the second day after inoculation, followed by an additional 4‐day incubation period. Metabolites were extracted from *A. alternata* mycelia and fermentation broths as described previously with minor modifications.^[^ ^44^ ^]^ After harvesting 1 g of *A. alternata* mycelia, the samples were homogenized in liquid nitrogen. The resulting mycelial powder was transferred to a 10 mL tube containing 5 mL of 50% methanol, and the pH was adjusted to 2.0 using 2 M HCl. The mixtures were shaken for 60 min at room temperature to extract metabolites. Following centrifugation at 5500 × *g* for 10 min, the supernatant was transferred to a new 10 mL tube. The crude extracts were lyophilized for 24 h using a vacuum lyophilizer (LGJ‐10, Xinyi Inc., Beijing, China) and redissolved in 1 mL of 50% methanol. The contents of metabolites were analyzed by UPLC‐MS as described above. Quantification of S‐TeA, TeA, AMHA, N‐AcAc‐AMHA, Ile, and N‐AcAc‐Ile was performed using the external standard method. Calibration curves for all quantified compounds exhibited linear ranges with correlation coefficients (*R* ^2^) above 0.999. Experiments were conducted in three independent biological replicates.

### RNA‐seq Library Construction and Data Analysis

Total RNA was extracted using the TRIzol reagent (Invitrogen), following the manufacturer's instructions. RNA purity was confirmed using a NanoDrop 2000 spectrophotometer (Thermo Fisher Scientific, MA, USA), and RNA integrity was assessed using the RNA 6000 Nano Kit with an Agilent 2100 Bioanalyzer (Agilent Technologies Inc., CA, USA). RNA concentration was determined with the Qubit RNA Assay Kit on a Qubit 3.0 fluorometer (Life Technologies Co., USA). RNA‐seq libraries were sequenced on the Illumina NovaSeq 6000 sequencing system at Biozeron Biotechnology Co., Ltd (Shanghai, China). Three independent biological replicates were used per treatment. Clean reads were obtained using SeqPrep software and aligned to the reference genome of *A. alternata* SRC1lrK2f (*Altal* 1 version) using HISAT2 (https://ccb.jhu.edu/software/hisat2/index.shtml), Raw counts and fragments per kilobase of transcript per million mapped (FPKM) values were calculated using the RSEM pipeline. Differential gene expression analysis was performed using the DESeq2, with genes meeting the criteria of *p* < 0.05 and an absolute value of log_2_‐transformed fold‐change (abs log_2_FC) ≥ 1 classified as differentially expressed. Gene Ontology (GO) enrichment analysis was conducted in R‐studio, with pathways considered significantly enriched at *p* < 0.05. Cytoscape software (version 3.10.0, USA), was used for protein interactions network visualization and quantification using the degree method.^[^ ^64^ ^]^

### RT‐qPCR Analysis

Total RNA was extracted from *A. alternata* mycelia using the TRIzol reagent (Invitrogen) and converted to cDNA with the PrimeScript RT Reagent Kit with gDNA Eraser (RR047A, TaKaRa, Tokyo, Japan) according to the manufacturer's protocol. RT‐qPCR was performed using TB Green Premix Ex Taq (Tli RNaseH Plus) (RR420A, TaKaRa) with all primers at a final concentration of 0.2 µM in an Eppendorf real‐time instrument (Mastercycler ep realplex^2^). The PCR program consisted of an initial denaturation at 95 °C for 30 s, followed by 40 cycles of 95 °C for 5 s and 60 °C for 30 s, and a final dissociation step at 95 °C for 15 s, 60 °C for 30 s, and 95 °C for 15 s. Relative gene expression levels were calculated using the 2^−ΔΔCT^ method, normalized to the expression of reference gene *actin*.^[^ ^65^ ^]^ Primer sequences used in this work were listed in Table S9, Supporting Information. All RT‐qPCR reactions consisted of four biological replicates per sample.

### Nontargeted Metabolome Profiling Analysis

Metabolite extraction was performed using the same method as described above. Samples were analyzed ay Beijing Novogene Co., Ltd. using UPLC‐MS/MS on a Vanquish UPLC system coupled with an Orbitrap Q Exactive HF mass spectrometer (Thermo Fisher, Germany). Six independent biological replicates were included. Raw data were processed using Compound Discoverer (version 3.1, Thermo Fisher, Germany) for peak alignment, peak picking, and quantitation. Adduct types [M−H]^−^ and [M+ H]^+^ were selected for negative‐ and positive‐ion modes, respectively. Peak intensities were normalized to the total spectral intensity. Molecular formula predictions based on additive ions, molecular ion peaks and fragment ions; qualitative analysis; and relative quantification were conducted using mzCloud (https://www.mzcloud.org), mzVaultand (https://mytracefinder.com/tag/mzvault) and MassList databases. Significant differential metabolites defined by abs log_2_FC ≥ 1, *p* < 0.05, and VIP ≥ 1. Metabolites were annotated and pathways identified using the Kyoto Encyclopedia of Genes and Genomes (KEGG; http://www.genome.jp/kegg/).

### Stable Isotope Labeling Experiments

To validate the key biosynthetic steps of S‐TeA, ^13^C_4_‐threonine was added to *A. alternata* fermentation broth (+^13^C_4_‐Thr) on the second day post‐inoculation. Parallel experiments with unlabeled threonine (natural‐abundance sample, −^13^C_4_‐Thr) were also conducted. The final concentration of ^13^C_4_‐threonine or unlabeled threonine was 20 mg L^−1^. Metabolite extraction and UPLC‐MS analysis were performed as above described.

Gene Cloning, Protein Heterologous Expression and Purification: Using genomic DNA of *A. alternata* as a template, genes coding for IPMS (CC77DRAFT_1 016 936), IPMDH (CC77DRAFT_955 795), and ISMD (CC77DRAFT_1 025 667), were optimized for *E. coli* expression. The resulting fragments of each gene were synthesized (GenScript Biotech Corporation, Nanjing, Jiangsu, China), and subcloned into the pET‐30a(+) vector via conventional ligation, generating the expression plasmids pET‐30a(+)‐IPMS, pET‐30a(+)‐IPMDH and pET‐30a(+)‐ISMD, each designed to express proteins with a C‐terminal polyhistidine tag. The NdeI and XhoI restriction sites were used for digestion and ligation. After validation by electrophoresis, the plasmids were transformed into *E. coli* TOP10 competent cells via heat shock. The transformed cells were plated onto LB medium containing kanamycin and incubated at 37 °C overnight. Selected monoclones were cultured overnight, and cells were harvested by centrifugation. The genetic constructs were verified by sequencing.

Protein expression and purification followed s standardized protocol for polyhistidine tagged proteins. *E. coli* BL21 (DE3) cells harboring the expression plasmids were grown LB medium supplemented with kanamycin at with shaking at 200 rpm until the OD_600_ reached 0.6. Cultures were transferred to a 16 °C shaker for 30 min to cool before inducing protein expression with 0.1 mM Isopropyl‐*β*‐D‐thiogalactopyranosid (IPTG). After overnight incubation at 16 °C, cells were harvested by centrifugation and resuspended in lysis buffer (20 mM Tris‐HCl (pH 8.0), 200 mM NaCl, 20 mM imidazole, 10% glycerol, and 1 mM Dithiothreitol (DTT)). Lysis was performed using a high‐pressure cell disruptor (Constant Systems) at 30 000 psi, and debris was removed by centrifugation (19 500 × *g* for 60 min at 4 °C. The lysate was applied to an affinity column for His‐tagged protein purification. The column was washed extensively with lysis buffer (20 column volumes), and proteins were eluted with elution buffer (lysis buffer containing 250 mM imidazole). Eluted fractions were further purified using a HiPrep 26/10 desalting column (GE healthcare, GE17‐5087‐01) pre‐equilibrated with desalting buffer (20 mM Tris‐HCl (pH 8.0), 200 mM NaCl, 10% glycerol and 1 mM DTT). Protein purity, typically above 95%, was confirmed by SDS‐PAGE with Coomassie Brilliant Blue R‐250 staining. Purified proteins were concentrated, flash frozen, and stored at −80 °C.

### In Vitro Enzymatic Activity Assays of IPMS, IPMDH, and ISMD

The enzymatic activity and kinetic parameters of IPMS were assessed following established protocols.^[^ ^42^ ^]^ Each 100 µL reaction mixture contained 1 mM acetyl‐CoA, 10 mM MgCl_2_, 10 mM potassium phosphate buffer (pH 7.5), 0.1 µM of IPMS and varying concentrations of OBA (10, 100, 500, 1000, 2000, or 4000 µM). Reactions were incubated at 30 °C for 1 h. A control experiment was conducted using the same conditions but without OBA to determine the baseline level of 2EMA production.

Similarly, the enzymatic activity and kinetic parameters of IPMDH were determined. Reaction mixtures (100 µL) contained 250 mM potassium phosphate (pH 7.0), 0.1 µM IPMDH, and 2EMA at concentrations of 10, 100, 500, 1000, 2000 or 4000 µM. Incubation was carried out at 30 °C for 1 h, with a control reaction excluding 2EMA to evaluate 3EMA formation.

For ISMD, assays were performed referring to as previous reference.^[^ ^66^ ^]^ Each 100 µL reaction mixture contained 0.1 µM ISMD, 1 mM NAD^+^, 5 mM MnCl_2_, 1 mM DTT, MOPS‐KCl buffer (25 mM MOPS and 100 mM KCl, pH 7.5), along with varying concentrations of 3EMA (10, 100, 500, 1000, 2000 or 4000 µM). Reactions were incubated at 30 °C for 1 h. A control experiment lacking 3EMA was included to assess OVA production.

To terminate reactions, 100 µL methanol was added in each mixture. Precipitated proteins were removed by centrifugation, and the supernatant was subjected to UPLC‐MS analysis. Quantifications of 2EMA, 3EMA, and OVA were based on external calibration curves, with linear ranges confirmed by correlation coefficients exceeding 0.999.

Each substrate concentration was tested in triplicate, and kinetic parameters, including maximum reaction rate (*V*_max_), Michaelis‐Menten constant (*K*_M_) and catalytic efficiency (*k*_cat_/*K*_M_). were calculated using the Enzyme Kinetic Module of GraphPad Prism version 9.5.1 (GraphPad software, San Diego, CA, USA).

### Statistical Analysis

All experiments were carried out with at least three independent biological replicates. Data were presented as mean ± SD. Statistical significance was determined through Student's *t*‐test and one‐way ANOVA analyses using GraphPad Prism version 9.5.1 (GraphPad software, San Diego, CA, USA). Asterisks indicate significant differences relative to the control (^*^ *p* < 0.05, ^**^ *p* < 0.01, ^***^ *p* < 0.001, ^****^ *p* < 0.0001).

## Conflict of Interest

The authors declare no conflict of interest.

## Author Contributions

S.C. conceived and designed the research; H.W., Y.G., Q.L., J.Z., Q.Z., M.Y. and Q.C. carried out experiments; S.C. and H.W. analyzed experimental data; S.Q. supervised the project; H.W., Y.G., and Q.L. wrote the manuscript; S.C. and B.E.V. reviewed, edited and validated the manuscript.

## Supporting information



Supporting Information

Supplemental Table 1

Supplemental Table 2

Supplemental Table 3

Supplemental Table 4

Supplemental Table 5

Supplemental Table 6

Supplemental Table 7

Supplemental Table 8

Supplemental Table 9

Supplemental Table 10

Supplemental Table 11

## Data Availability

The data that support the findings of this study are available in the supplementary material of this article.
