# Supplementary material for: Discovery, Herbicidal Activity and Biosynthesis of a Novel Natural Tetramic Acid from Alternaria Species
Source: Adv Sci (Weinh). 2025 Apr 25;12(21):2416188. doi: 10.1002/advs.202416188 (PMC12140367; doi:10.1002/advs.202416188)
Supplement: Supplementary file 1 — Supporting Information [file ADVS-12-2416188-s009.docx]

Supplemental Information

**Discovery, Herbicidal Activity and Biosynthesis of a Novel Natural Tetramic Acid from *Alternaria Species***

*He Wang, Yanjing Guo, Qing Liu, Jing Zhang, Qianlong Zhang, Mingying Yang, Qizhen Chen, Sheng Qiang, Bernal E. Valverde, and Shiguo Chen**

H. Wang, Y. Guo, Q. Liu, J. Zhang, Q. Zhang, M. Yang, Q. Chen, S. Qiang, B. E. Valverde, S. Chen

State Key Laboratory of Agricultural and Forestry Biosecurity, College of Life Sciences

Nanjing Agricultural University

Nanjing 210095, P. R. China

E-mail: chenshg@njau.edu.cn

B. E. Valverde

Research and Development in Tropical Agriculture

Alajuela 4050, Costa Rica

**Table of Contents**

[1 Supplemental Methods 1](#_Toc195258294)

[1.1 Modeling of Ile or AMHA in AaTAS1 1](#_Toc195258295)

[1.2 Modeling of TeA or S-TeA in AaMFS1 1](#_Toc195258296)

[1.3 Syntheses schemes was used in this study 1](#_Toc195258297)

[1.4 ^1^H NMR and ^13^C NMR spectra 7](#_Toc195258298)

[2 Supplemental Figures 15](#_Toc195258299)

[Figure S1. 15](#_Toc195258300)

[Figure S2. 16](#_Toc195258301)

[Figure S3. 17](#_Toc195258302)

[Figure S4. 18-19](#_Toc195258303)

[Figure S5. 19](#_Toc195258304)

[Figure S6. 20](#_Toc195258305)

[Figure S7. 21](#_Toc195258306)

[Figure S8. 22](#_Toc195258307)

[Figure S9. 23](#_Toc195258308)

[Figure S10. 24](#_Toc195258309)

[Figure S11. 25](#_Toc195258310)

[Figure S12. 26](#_Toc195258311)

[3 Reference 27](#_Toc195258312)

[4. Supporting information 27](#_Toc195258313)

# 1 Supplemental Methods

## 1.1 *Modeling of* *Ile or AMHA in AaTAS1*: As the crystal structure of *Aa*TAS1 protein is not available, a high-resolution homology model of the *Aa*TAS1 of *A. alternata* was built by Alphafold3 (https://deepmind.google/technologies/alphafold/alphafold-server/). The conserved domain structure was analyzed through the Simple Modular Architecture Research Tool (SMART, https://smart.embl.de/). The docking program Discovery Studio (version 2019, BIOVIA, USA) was used to explore the specific action target of Ile or AMHA in *Aa*TAS1. The amino acid residues including His541, Arg544, Met564, Thr678 and Phe679 in A domain of *Aa*TAS1was defined as the binding site for Ile or AMHA according to the results of automate the preliminary docking process with the LibDock tool of Discovery Studio. The accurate molecular docking was performed with the CDocker tool of Discovery Studio.

## 1.2 *Modeling of TeA or S-TeA in* *AaMFS1*: As the crystal structure of *Aa*MFS1 protein is not available,^[1]^ a high-resolution homology model of the *Aa*MFS1 of *A. alternata* was built by Alphafold3 (https://deepmind.google/technologies/alphafold/alphafold-server/). The conserved domain structure was analyzed through the Simple Modular Architecture Research Tool (SMART, https://smart.embl.de/). The docking program Discovery Studio (version 2019, BIOVIA, USA) was used to explore the specific action target of TeA or S-TeA in *Aa*MFS1. The amino acid residues Ser28, Leu32, Phe96, Thr126 and Val189 in *Aa*MFS1 was defined as the binding site for TeA or S-TeA according to the results of automate the preliminary docking process with the LibDock tool of Discovery Studio. The accurate molecular docking was performed with the CDocker tool of Discovery Studio.

## 1.3 Syntheses schemes was used in this study


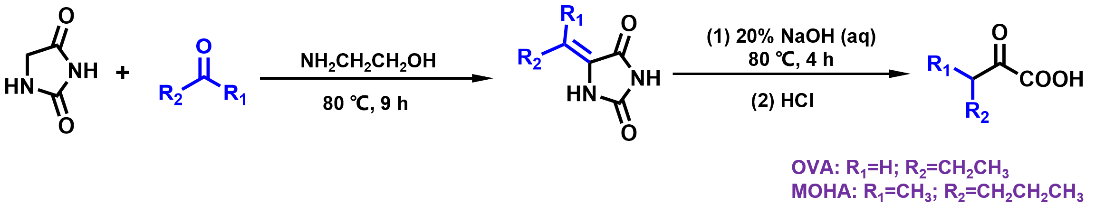


**Scheme 1.** The procedure for the synthesis of 2-oxovaleric acid (OVA) and 3-methyl-2-oxohexanoic acid (MOHA)

As it was shown in **Scheme 1**, hydantoin (10.0 g, 0.1 mol), propanal (7.6 g, 0.13 mol) or 2-pentanone (11.2 g, 0.13 mol) and H_2_O (12 mL) were combined together in a three-neck glass flask (250 mL) and stirred for 5 min at room temperature. And then 2-aminoethanol (4.8 g, 0.08 mol) was added dropwise in the mixture and stirred for 9 h at 80℃. The white solid was precipitated till the temperature of mixture was cool down. The white solid was washed with H_2_O and dried to get 2-propylhydantoin or 2-pentylidenehydantoin. 2-propylhydantoin (14.0 g, 0.1 mol) or 2-pentylidenehydantoin (16.8 g, 0.1 mol) and 20% NaOH aqueous (100 mL, 0.5 mol) was combined together in a three-neck glass flask (250 mL) and stirred for 4 h at 80℃. Subsequently, HCl (6 M) was slowly added in the mixture to adjust the pH to 9 and then filtered, and concentrated under vacuum to get residue. 50 mL H_2_O was added to dissolve the residue after concentrated, and 50 mL methanol was added dropwise and stirred. The precipitate was filtered and the filtrate was concentrated under vacuum at 65℃ in a rotary evaporator (EYELA, Japan) to offer the 2-oxovaleric acid or 3-methyl-2-oxohexanoic acid.

**2-oxovaleric acid (OVA):**


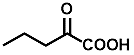


White solid, 97 % yield. ^1^H NMR (500 MHz, D_2_O) δ 4.57 (s, 2H, C*H*_2_CO), 1.47–1.20 (m, 2H, C*H*_2_CH_3_), 0.80 (t, *J* =15 Hz, 3H, CH_2_C*H*_3_). ^13^C NMR (125 MHz, D_2_O) *δ* = 206.96 (*C*OOH), 160.48 (CH_2_*C*O), 25.47 (*C*H_2_CO), 21.32 (*C*H_2_CH_3_), 14.37 (CH_2_*C*H_3_). HRMS (ESI) *m/z* calcd. for C_5_H_10_O [M+H]^+^: 117.0552, found: 117.0532.

**3-methyl-2-oxohexanoic acid (MOHA):**

White solid, 98 % yield. ^1^H NMR (500 MHz, D_2_O) *δ* 2.87–2.83 (m, 1H, C*H*CH_3_), 1.49–1.17 (m, 4H, C*H*_2_C*H*_2_CH_3_), 0.92 (d, *J* =10 Hz, 3H, CH_2_CH_2_C*H*_3_), 0.72 (t, *J* =5 Hz, 3H, CHC*H*_3_). ^13^C NMR (125 MHz, D_2_O) *δ* = 211.53 (*C*OOH), 160.48 (CH*C*O), 41.99 (*C*HCH_3_), 33.57 (*C*H_2_CH_2_CH_3_), 19.60 (CH_2_*C*H_2_CH_3_), 14.19 (CH*C*H_3_), 13.32 (CH_2_CH_2_*C*H_3_). HRMS (ESI) *m/z* calcd. for C_7_H_12_O_3_ [M+H]^+^: 145.0865, found: 145.0901.


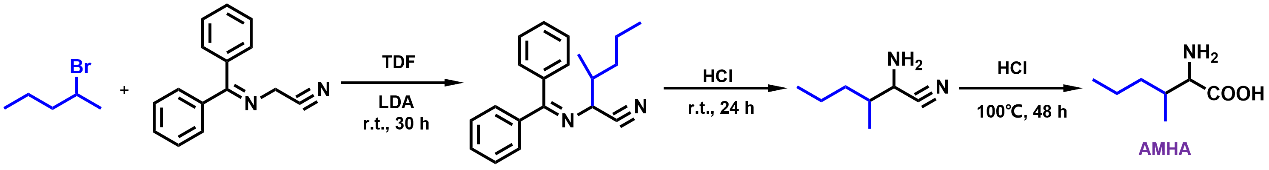


**Scheme 2.** The procedure for the synthesis of 2-amino-3-methylhexanoic acid (AMHA).

The AMHA was prepared according to Qiang et al (**Scheme 2**).^[2]^ The catalyst lithium diisopropylamide (LDA, 3 mL, 6 mmol) was added to 30 mL tetrahydrofuran (THF) solution of N-(diphenylmethylene)aminoacetonitrile (1.1 g, 5 mmol) in a three-neck glass flask (250 mL) and stirred for 5 min, and then 0.74 mL 2-bromopentane was slowly added under a given nitrogen atmosphere at 0℃. The mixture was stirred for 30 h at room temperature, and 20 mL saturated aqueous NH_4_Cl (7 M) was added to stop the reaction. The organic layer was dried over anhydrous Na_2_SO_4_, filtered and evaporated under vacuum at 65℃ in a rotary evaporator (EYELA, Japan). The crude extract was purified by column chromatography on silica gel (200–300 mesh, Qingdao Marine Chemical Co. Ltd., Qingdao, China) (petroleum ether: EtOAc = 20: 1) to afford yellow-green oil product of 2-((diphenylmethylene)amino)-3-methylhexanenitrile Subsequently, the oil product 2-((diphenylmethylene)amino)-3-methylhexanenitrile (0.87 g, 3 mmol) was dissolved in 20 mL ether in a three-neck glass flask and 4 mL HCl (1 M) was added. The mixture was stirred for 24 h at room temperature and washed with ether (2 × 10 mL) in a separating funnel, and the aqueous layer was evaporated under vacuum at 100℃ in a rotary evaporator (EYELA, Japan) to leave white solid of 2-amino-3-methylhexanenitrile was dissolved in 10 mL HCl (6 M) and stirred for 48 h at 100℃ in a three-neck glass flask (250 mL). The mixture was cooled down to room temperature and washed with ether (2 × 10 mL) in a separating funnel, and the aqueous layer was evaporated under vacuum at 100℃ in a rotary evaporator (EYELA, Japan) to get the 2-amino-3-methylhexanoic acid.

**2-amino-3-methylhexanoic acid (AMHA):**

White solid, 91 % yield. ^1^H NMR (500 MHz, D_2_O) *δ* 3.81–3.78 (m, 1H, C*H*NH), 2.11–1.98 (m, 1H, C*H*CH_3_); 1.22–1.20 (m, 2H, C*H*_2_C*H*_2_CH_3_), 1.22–1.19 (m, 2H, C*H*_2_CH_2_CH_3_), 1.14–1.10 (m, 2H, CH_2_C*H*_2_CH_3_), 0.83 (d, *J* =20 Hz, 3H, CHC*H*_3_), 0.70 (t, *J* =5 Hz, 3H, CH_2_CH_2_C*H*_3_). ^13^C NMR (125 MHz, D_2_O) *δ* = 171.89 (*C*OOH), 56.77 (*C*HNH), 37.42 (*C*HCH_3_), 33.19 (*C*H_2_CH_2_CH_3_), 16.09 (CH_2_*C*H_2_CH_3_), 14.11 (CH*C*H_3_), 12.13 (CH_2_CH_2_*C*H_3_). HRMS (ESI) *m*/*z* calcd. for C_7_H_15_NO_2_ [M+H]^+^: 146.1181, found: 146.1167.


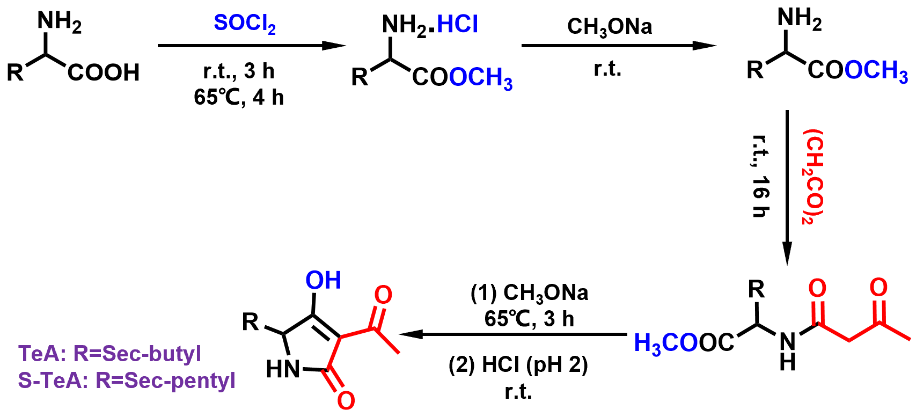


**Scheme 3.** The procedure for the synthesis of 3-acetyl-5-sec-butyl-4-hydroxy-pyrrolidine-2-one (TeA) and 3-acetyl-5-sec-penyl-4-hydroxy-pyrrolidine-2-one (S-TeA).

TeA or S-TeA were synthesized by a straightforward five-step synthesis approach including esterification, neutralization, acidylation, cyclization and acidification as previously described with some modifications (**Scheme 3**).^[3]^ After SOCl_2_ (1.42 *g*, 12 mmol) was added in 50 mL CH_3_OH in a three-neck glass flask (250 mL) and stirred for 1 h at 0℃, the starting material *L*-isoleucine (1.31 g, 10 mmol) or 2-amino-3-methylhexanoic acid (1.45 g, 10 mmol) was added. Subsequently, the mixture was stirred for 3 h at room temperature, and then heated for 4 h at 65℃. The solvent in the reaction mixture was evaporated under vacuum at 65℃ in a rotary evaporator (EYELA, Japan) to get the esterification product, which was re-dissolved in 50 mL CH_3_OH in a three-neck glass flask (250 mL). CH_3_ONa (0.11 g sodium metal in 10 mL CH_3_OH) was added in the CH_3_OH solution of the esterification product to get the neutralization product of *L*-isoleucine methyl ester or 2-amino-3-methylhexanoic acid methyl ester. And then, (CH_2_CO)_2_ (0.42 g, 5 mmol) was added and stirred for 16 h at room temperature. After the solvent in the reaction mixture was evaporated under vacuum at 65℃ in a rotary evaporator (EYELA, Japan), the crude extract was dissolved in 50 mL EtoAc and washed, respectively, with HCl (1 M, 2 × 5 mL) and aqueous NaHCO_3_ (0.5 M, 2 × 5 mL) in a separating funnel. The organic layer was dried over anhydrous Na_2_SO_4_, filtered and evaporated at 65℃ in a rotary evaporator (EYELA, Japan) to get the acidylation product of N-acetoacetyl-*L*-isoleucine methyl ester or N-acetoacetyl-2-amino-3-methylhexanoic acid methyl ester. After the acidylation product (5 mmol) was re-dissolved in 50 mL CH_3_OH, CH3ONa (0.11 *g* sodium metal in 10 mL CH_3_OH) was added and stirred for 3 h at 65℃. The solvent in the reaction mixture was evaporated under vacuum at 65℃ in a rotary evaporator (EYELA, Japan) to get the cyclization product. Finally, the cyclization product was dissolved in 50 mL water and acidified with HCl (6 M) at pH 2. After twenty mL of ether was added in the mixture in a separating funnel and shaken at room temperature, and the organic layer was dried over anhydrous Na_2_SO_4_, filtered and evaporated at 65℃ in a rotary evaporator (EYELA, Japan) to get TeA or S-TeA.

**3-Acetyl-5-sec-butyl-4-hydroxy-pyrrolidine-2-one (TeA)：**

Light brown oil, 90 % yield. ^1^H NMR (500 MHz, chloroform-*d*) *δ* 10.41 (br, 1H, CHO*H*), 7.83–7.68 (m, 1H, CHN*H*), 3.85–3.70 (m, 1H, C*H*NH), 2.36 (s, 3H, COC*H*_3_), 1.90–1.85 (m, H, C*H*CH_3_), 1.28–1.14 (m, 2H, C*H*_2_CH_3_), 0.92 (d, *J* =10 Hz, 3H, CHC*H*_3_), 0.79 (t, *J* =10 Hz, 3H, CH_2_C*H*_3_). ^13^C NMR (125 MHz, chloroform-*d*) *δ* 195.94 (*C*O CH_3_), 184.43 (*C*HOH), 175.82 (*C*ONH), 102.55 (*C*CO), 67.41 (*C*HNH), 36.95 (*C*HCH_3_), 23.57 (*C*OCH_3_), 15.77 (CH*C*H_3_), 11.73 (CH_2_*C*H_3_). HRMS (ESI) *m/z* calcd. for C_10_H_15_NO_3_ [M+H]^+^: 198.1130, found: 198.1140.

**3-acetyl-5-sec-penyl-4-hydroxy-pyrrolidine-2-one (S-TeA):**

Orange solid, 90 % yield. ^1^H NMR (500 MHz, methanol-*d_4_*) *δ* 8.50 (br, 1H, CHN*H*), 3.72 (s, 1H, C*H*NH), 2.27 (s, 3H, COC*H*_3_), 1.85 (s, H, C*H*CH_3_), 1.27–1.20 (m, 2H, C*H*_2_CH_2_CH_3_), 1.16–1.10 (m, 2H, CH_2_C*H*_2_CH_3_), 0.80 (t, *J* =10 Hz, 3H, CH_2_C*H*_3_), 0.59 (d, *J* =5 Hz, 3H, CHC*H*_3_). ^13^C NMR (125 MHz, methanol-*d_4_*) *δ* 196.16 (*C*OCH_3_), 184.62 (*C*HOH), 175.56 (*C*ONH), 102.64 (*C*CO), 67.42 (*C*HNH), 36.12 (*C*H_2_CH_2_CH_3_), 34.35 (*C*HCH_3_), 30.35 (CO*C*H_3_), 20.26 (CH_2_*C*H_2_CH_3_), 14.38 (CH*C*H_3_), 13.59 (CH_2_*C*H_3_). HRMS (ESI) *m/z* calcd. for C_11_H_17_NO_3_ [M+H]^+^: 212.1290, found: 212.1289.


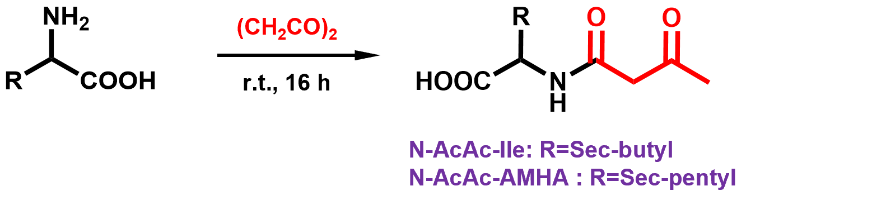


**Scheme 4.** The procedure for the synthesis of N-acetoacetyl-isoleucine (N-AcAc-Ile) and N-acetoacetyl-2-amino-3-methylhexanoic acid (N-AcAc-AMHA).

As it was shown in **Scheme 4**, *L*-isoleucine (1.31 g, 10 mmol) or 2-amino-3-methylhexanoic acid (1.45 g, 10 mmol) and H_2_O (20 mL) were combined together in a three-neck glass flask (250 mL) and (CH_2_CO)_2_ (0.42 g, 5 mmol) was added dropwise at 0℃, and then stirred for 16 h at room temperature. After the solvent in the reaction mixture was evaporated under vacuum at 100℃ in a rotary evaporator (EYELA, Japan), the crude extract was dissolved in 50 mL EtoAc and washed, respectively, with HCl (1 M, 2 × 5 mL) and aqueous NaHCO_3_ (0.5 M, 2 × 5 mL) in a separating funnel. The organic layer was dried over anhydrous Na_2_SO_4_, filtered and evaporated at 65℃ in a rotary evaporator (EYELA, Japan) to get the acidylation product of N-acetoacetyl-isoleucine or N-acetoacetyl-2-amino-3-methylhexanoic acid.

**N-acetoacetyl-isoleucine (N-AcAc-Ile)：**

Yellow liquid, 95 % yield. ^1^H NMR (500 MHz, chloroform-*d*) *δ* 10.30 (br, 1H, COO*H*), 7.76(s, 1H, CHN*H*), 4.51–4.50 (m, 1H, C*H*NH), 3.48 (s, 2H, COC*H*_2_CO), 2.19 (s, 3H, COCH_3_), 2.04–1.82 (m, 1H, C*H*CH_3_), 1.43–1.13 (m, 2H, C*H*_2_CH_3_), 0.87 (t, *J* =10 Hz, 3H, CH_2_C*H*_3_), 0.85 (d, *J* =20 Hz, 3H, CHC*H*_3_). ^13^C NMR (125 MHz, chloroform-*d*) *δ* 204.58 (*C*OCH_3_), 174.57 (*C*OOH), 167.04 (*C*ONH), 56.85 (*C*HNH), 49.13 (*C*H_2_CO), 37.43 (*C*HCH_3_), 30.78 (CO*C*H_3_), 24.99(*C*H_2_CH_3_), 15.54 (CH*C*H_3_), 11.62 (CH_2_*C*H_3_). HRMS (ESI) *m/z* calcd. for C_10_H_17_NO_4_ [M+H]^+^: 216.1229, found: 216.1225.

**N-acetoacetyl-2-amino-3-methylhexanoic acid (N-AcAc-AMHA)：**

Yellow liquid, 95 % yield. ^1^H NMR (500 MHz, chloroform-*d*) *δ* 7.62(br, 1H, CHN*H*), 4.65-4.54 (m, 1H, C*H*NH), 3.50 (s, 2H, COC*H*_2_CO), 2.27 (s, 3H, COCH_3_), 2.08–2.00 (m, 1H, C*H*CH_3_), 1.43–1.36 (m, 2H, C*H*_2_CH_2_CH_3_), 1.28–1.22 (m, 2H, CH_2_C*H*_2_CH_3_), 0.94 (t, *J* =10 Hz, 3H, CH_2_C*H*_3_), 0.89 (d, *J* =10 Hz, 3H, CHC*H*_3_). ^13^C NMR (125 MHz, chloroform-*d*) *δ* 204.30 (*C*OCH_3_), 172.12 (*C*OOH), 165.58 (*C*ONH), 56.90 (*C*HNH), 49.51 (*C*H_2_CO), 35.54 (*C*H_2_CH_2_CH_3_), 34.43 (*C*HCH_3_), 31.00 (CO*C*H_3_), 20.24 (CH_2_*C*H_2_CH_3_), 16.10 (CH*C*H_3_), 14.14 (CH_2_*C*H_3_). HRMS (ESI) *m/z* calcd. for C_11_H_19_NO_4_ [M+H]^+^: 230.1314, found: 230.1377.

## 1.4 ^1^H NMR and ^13^C NMR spectra

In this study, ^1^H and ^13^C NMR spectra were recorded on a JEOL (500 MHz for ^1^H and 125 MHz for ^13^C respectively) instrument (JEOL Resonance Inc. Tokyo, Japan), and were internally referenced to residual solvent signals, chloroform-*d* referenced at *δ* 7.26 and 77.00 ppm, DMSO-*d*_6_ referenced at *δ* 2.50 and 39.80 ppm, D_2_O referenced at 4.80 ppm, methanol-*d*_4_ referenced at 3.31 and 49.00 ppm. Data for ^1^H was reported as follows: chemical shift (*δ* ppm), integration, multiplicity (s referred singlet, d r referred doublet, t referred triplet, m referred multiplet), broad peaks (br), coupling constant (Hz) and assignment. Data for ^13^C NMR was reported in terms of chemical shift (*δ* ppm), multiplicity and coupling constant (Hz).

^1^H NMR spectrum of OVA

^13^C NMR spectrum of OVA

^1^H NMR spectrum of MOHA

^13^C NMR spectrum of MOHA

^1^H NMR spectrum of AMHA

^13^C NMR spectrum of AMHA

^1^H NMR spectrum of TeA

^13^C NMR spectrum of TeA

^1^H NMR spectrum of S-TeA

^13^C NMR spectrum of S-TeA

^1^H NMR spectrum of N-AcAc-Ile

^13^C NMR spectrum of N-AcAc-Ile

^1^H NMR spectrum of N-AcAc-AMHA

^13^C NMR spectrum of N-AcAc-AMHA

# 2 Supplemental Figures

**Figure S1.** **The 2D NMR spectra of Compound S**. A) The ^1^H-^1^H-COSY spectrum of Compound S. B) The HMBC spectrum of Compound S.

**Figure S2. The HPLC-MS chromatograms and mass spectra of natural S-TeA produced by *M. oryzae* A), *A. alternata* f. sp. *Lycopersici* B) and *A. brassicicola* C) cultivated for 7 d in the fermentation broth**.

**Figure S3. Phytotoxicity of natural S-TeA (Nat-S-TeA) and S-TeA diastereomers to *A. adenophora* leaves at increasing concentrations**. A) Diagram of placement of compounds to detached leaves in puncture assay and phytotoxic responses to TeA and the chemical (Chem) and natural (Nat-) forms of S-TeA. B) Diagram as in (A) and phytotoxicity of TeA, Chem-S-TeA or Nat-S-TeA to *A. adenophora*. C) Diagram of placement of compounds to detached leaves in puncture assay and phytotoxic responses to Nat-S-TeA, (5*S*, 6*S*)-S-TeA, (5*S*, 6*R*)-S-TeA, (5*R*, 6*S*)-S-TeA or (5*R*, 6*R*)-S-TeA. D) Diagram as in (C) and phytotoxicity of Nat-S-TeA, (5*S*, 6*S*)-S-TeA, (5*S*, 6*R*)-S-TeA, (5*R*, 6*S*)-S-TeA or (5*R*, 6*R*)-S-TeA to *A. adenophora*. The experiments consisted of ten independent biological replicates with similar results.

**Figure S4.** **Chl *a* fluorescence rise kinetics of leaf discs of *A. adenophora* treated with different S-TeA diastereomers**. The fluorescence O-J-I-P curves (O-, J-, I-, and P-step refers to the fluorescence at 20 µs, 2 ms, 30 ms, and the peak P of O-J-I-P, respectively) of leaf discs of *A. adenophora* (left) and the kinetics double-normalized by F_O_ (minimal fluorescence) and F_M_ (maximal fluorescence) (right) treated S-TeA diastereomers at increasing concentrations: A) (5*S*, 6*S*)-S-TeA, B) (5*S*, 6*R*)-S-TeA, C) (5*R*, 6*S*)-S-TeA, D) (5*R*, 6*R*)-S-TeA. E) comparison of Chl *a* fluorescence rise kinetics of leaf discs of *A. adenophora* treated with S-TeA diastereomers at 200 μM. Each data point is the average of three independent biological replicates with ten different leaf samples for each replication.

**Figure S5. Model simulation of S-TeA diastereomers binding to the Q_B_ binding site of D1 protein of *A. adenophora*.** A) The 2D diagram of (5*S*, 6*S*)-S-TeA binding environment in the Q_B_ binding site. B) 2D diagram of the (5*S*, 6*R*)-S-TeA binding environment at the Q_B_ binding site. C) 2D diagram of the (5*R*, 6*S*)-S-TeA binding environment at the Q_B_ binding site. D) 2D diagram of the (5*R*, 6*R*)-S-TeA binding environment at the Q_B_ binding site. Carbon atoms are shown in grey, nitrogen atoms in blue, oxygen in red. The possible hydrogen bond is indicated by a dashed line.

**Figure S6. Content of S-TeA, TeA and their key intermediate metabolites after adding different precursors**. Content of S-TeA, AMHA and N-AcAc-AMHA and the correlations between AMHA or N-AcAc-AMHA and S-TeA in *A. alternata* cultures after 6 days of growth, with A) aspartic acid, B) arginine or C) isoleucine supplementation occurring on the second day following fungus inoculation. D) Content of TeA, Ile or N-AcAc-Ile and the correlations between Ile or N-AcAc-Ile and TeA in *A. alternata* cultures after 6 d of growth, with threonine supplementation occurring on the second day following fungus inoculation. Data shown are mean values ± SD of three independent biological replicates.

**Figure S7.** **Differential expression gene statistics of** ***A. alternata* fed with threonine.** A) The volcano plots of differentially expressed genes. B) Multigroup heat map with normalized FPKM and bubble chart of Gene Ontology (GO) enrichment analysis of genes in cluster 4 or cluster 7.

**Figure S8. The labeling of TeA synthesized from uniformly ^13^C labeled threonine (^13^C_4_-Thr) in *A. alternata*.** A) Routes for biosynthesis of TeA and its predicted labeling from ^13^C_4_-Thr. B) Mass spectra of key metabolites synthesized from unlabeled threonine (−^13^C_4_-Thr) in *A. alternata*. C) Mass spectra of key metabolites synthesized from ^13^C_4_-Thr (+^13^C_4_-Thr) in *A. alternata*. Unlabeled and newly synthesized compounds from ^13^C_4_-Thr labeled are shown as black and blue lines, respectively. The response of the most abundant peak in each graph was set to 100%. Thr, threonine; OBA, 2-oxobutanoic acid; Ac-CoA, acetyl-coenzyme A; AHBA, 2-acetyl-2-hydroxybutyric acid; DHMPA, 2,3-dihydroxy-3-methylpentanoic acid; MOPA, 3-methyl-2-oxopentanoic acid; AKG, *α*-ketoglutaric acid; Ile, isoleucine; TD, threonine deaminase; ALS, acetolactate synthase; KARI, ketol-acid reductoisomerase; DHAD, dihydroxy acid dehydratase; BCAT, branched-chain amino acid aminotransferase; NRPS, non-ribosomal peptide synthetases.

**Figure S9. HPLC-MS chromatogram of six key metabolites in TeA biosynthetic pathway.** A) The HPLC-MS chromatogram of standard. B) The HPLC-MS chromatogram of extracts from mycelia of *A. alternata.*

**Figure S10.** **HPLC-MS chromatogram of nine key metabolites in S-TeA biosynthetic pathway**. A) The HPLC-MS chromatogram of standard. B) The HPLC-MS chromatogram of extracts from mycelia of *A. alternata.*

**Figure S11.** **The docking of possible binding site of *Aa*TAS1 of *A. alternata* with IIe or AMHA.** A) The domain structure of *Aa*TAS1. B) The 3D-model of *Aa*TAS1. Here, C (condensation) domain, A (adenylation) domain, PCP (peptidyl carrier protein) domain, and KS (ketosynthase) domain were colored in yellow, golden, green and pink, other area was colored in blue. C) Docked poses of Ile binding to *Aa*TAS1. D) Surface representation of the *Aa*TAS1 with bound Ile. E) Docked poses of AMHA binding to *Aa*TAS1. F) Surface representation of the *Aa*TAS1 with bound AMHA. Here, carbon atoms are shown in grey, nitrogen atoms in blue, oxygen in red and hydrogen atoms in white. The possible hydrogen bond is indicated by dashed line.

**Figure S12.** **The docking of possible binding site of *Aa*MFS1 of *A. alternata* with TeA or S-TeA.** A) Expression level of the *AaMFS1* gene of *A. alternata* (normalized to *ACTIN*) in –Thr or +Thr groups cultured for 1, 2, 3 and 6 d, respectively. B) The domain structure (above) and 3D-model (below) of *Aa*MFS1. Here, MFS superfamily domain was color in golden, other area was colored in blue. C) Docked poses of TeA inside the binding site of *Aa*MFS1. D) Docked poses of S-TeA inside the binding site of *Aa*MFS1. E) Hydrogen bond interactions for TeA binding to *Aa*MFS1. F) Hydrogen bond interactions for S-TeA binding to *Aa*MFS1. Here, carbon atoms are shown in grey, nitrogen atoms in blue, oxygen in red and hydrogen atoms in white. The possible hydrogen bond is indicated by dashed line.

# 3 Reference

1. F. Sun, X. Cao, D. Yu, D. Hu, Z. Yan, Y. Fan, Y. Wang, A. Wu, *Mol. Plant-Microbe Interact.* **2022**, *35*, 416.
2. S. Qiang, Q. Zhang, Z. Wang, H. Zhu, S. Chen, CN108358797A, **2018**.
3. C. Yang, S. Qiang, L. Huang, P. Zhang, Z. Zhu, ZL200610038765.X, **2008**.

# 4. Supporting information

Additional supporting information including Supplemental Tables can be found online in supporting information section at the end of the article.

**Supplemental Tables**

**Table S1. Structures, molecular solvent accessible surface area (Molecular-SASA) and possible interactions for (5*S*, 6*S*)-S-TeA, (5*S*, 6*R*)-S-TeA, (5*R*, 6*S*)-S-TeA and (5*R*, 6*R*)-S-TeA binding to the D1 protein of *A. adenophora*.**

**Table S2.** **Differentially expressed genes statistics of *A. alternata* cultured for different days or fed threonine on the second day after inoculation.**

**Table S2-1.** List of upregulated and downregulated genes of *A. alternata* in −Thr 3 d/ −Thr 1 d group.

**Table S2-2.** List of upregulated and downregulated genes of *A. alternata* in −Thr 6 d/ −Thr 1 d group.

**Table S2-3.** List of upregulated and downregulated genes of *A. alternata* in +Thr 3 d/ −Thr 1 d group.

**Table S2-4.** List of upregulated and downregulated genes of *A. alternata* in +Thr 6 d/ −Thr 1 d group.

**Table S2-5.** List of upregulated and downregulated genes of *A. alternata* in +Thr 3 d/ −Thr 3 d group.

**Table S2-6.** List of upregulated and downregulated genes of *A. alternata* in +Thr 6 d/ −Thr 6 d group.

**Table S3. Gene clustering analysis and GO term enrichment analysis of *A. alternata* in groups of** −**Thr 1 d,** −**Thr 3 d,** −**Thr 6 d, +Thr 3 d and +Thr 6 d.**

**Table S3-1.** List of genes in *A. alternata* in the groups of −Thr 1 d, −Thr 3 d, −Thr 6 d, +Thr 3 d and +Thr 6 d. The total gene expression patterns were normalized based on fragments per kilobase of exon per million mapped reads (FPKM) and divided into ten clusters

**Table S3-2.** List of enriched GO terms in the biological process category of the 1505 genes of cluster 3 listed in Table S3-1.

**Table S3-3.** List of enriched GO terms in the biological process category of the 753 genes of cluster 7 listed in Table S3-1.

**Table S4. The up-regulated differentially expressed genes statistics of *A. alternata* cultured in +Thr 3 d/** −**Thr 3 d and +Thr 6 d/** −**Thr 6 d groups.**

**Tabel S4-1.** List of 1569 genes that were only upregulated in +Thr 3 d/−Thr 3 d group not in +Thr 6 d/−Thr 6 d group of *A. alternata*.

**Table S4-2.** List of 407 genes that only were upregulated in +Thr 6 d/−Thr 6 d group not in +Thr 3 d/−Thr 3 d group of *A. alternata*.

**Table S4-3.** List of 310 common upregulated genes of *A. alternata* in +Thr 3 d/−Thr 3 d and +Thr 6 d/−Thr 6 d groups.

**Table S4-4.** List of enriched GO terms in the biological process category of the 310 genes listed in Table S4-3.

**Table S5. List of genes belonging to the amino acid biosynthesis, small molecule biosynthesis and NRPS and PKS, in** −**Thr 3 d/** −**Thr 1 d,** −**Thr 6 d/** −**Thr 1 d, +Thr 3 d/** −**Thr 1 d, +Thr 6 d/** −**Thr 1 d, +Thr 3 d/** −**Thr 3 d and +Thr 6 d/** −**Thr 6 d groups.**

**Table S6. Connectivity and closeness centrality degrees of twenty-three enzymes belonging to amino acid biosynthesis pathway, small molecule biosynthesis or nonribosomal peptide synthetases (NRPS) and polyketide synthase (PKS).**

**Table S7. Differential metabolite statistics of *A. alternata* in** −**Thr 1 d,** −**Thr 6 d and +Thr 6 d groups.**

**Table S7-1.** List of metabolites of *A. alternata* in −Thr 1 d, −Thr 6 d and +Thr 6 d groups. The intensity of each metabolite was normalized and divided into eight clusters.

**Table S7-2.** List of KEGG enrichment analysis of the metabolites in each cluster listed in Table S7-1 with a significance of *p*-value < 0.05.

**Table S7-3.** List of key up-regulated metabolites of S-TeA and TeA biosynthetic pathway of *A. alternata* in +Thr 6 d/ −Thr 6 d group.

**Table S8. Structure and possible interactions for Ile and AMHA binding to the *Aa*TAS1 protein.**

**Table S9. List of genes and primer sequences used for the gene expression analysis by qRT-PCR in this study.**

**Table S10. List of genes in *A. alternata* cultured for 1 d (−Thr 1 d), 3 d (−Thr 3 d) and 6 d (−Thr 6 d) after inoculation or cultured for 3 d (+Thr 3 d) and 6 d (+Thr 6 d) which fed threonine on the second day after inoculation based on RNA-Seq data.**

**Table S11. List of metabolites in *A. alternata* cultured for 1 d (−Thr 1 d) and 6 d (−Thr 6 d) after inoculation or cultured for 6 d (+Thr 6 d) which fed threonine on the second day after inoculation based on nontargeted metabolome data.**
