# Supplementary material for: Discovery, Herbicidal Activity and Biosynthesis of a Novel Natural Tetramic Acid from Alternaria Species
Source: Adv Sci (Weinh). 2025 Apr 25;12(21):2416188. doi: 10.1002/advs.202416188 (PMC12140367; doi:10.1002/advs.202416188)
Supplement: Supplementary file 2 — Supplemental Table 1 [file ADVS-12-2416188-s010.docx]

**Table S1.** Structures**,** molecular solvent accessible surface area (Molecular-SASA) and possible interactions for (5*S*, 6*S*)-S-TeA, (5*S*, 6*R*)-S-TeA, (5*R*, 6*S*)-S-TeA and (5*R*, 6*R*)-S-TeA binding to the D1 protein of *Ageratina adenophora*.

| Compound | Chemical Structure | Molecular-SASA  (Å^2^) | Bonding donors | Bonding acceptors | Interactions | Bound distance (Å) | Interaction energy  (kcal mol^−1^) |
| --- | --- | --- | --- | --- | --- | --- | --- |
| (5*S*, 6*S*)-S-TeA | 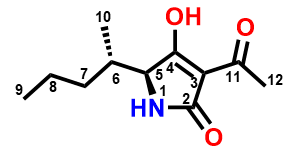 | 394.90 | D1-His252  D1-Phe255  **D1-Gly256 NH**  D1-Phe260  D1-Gln261  D1-Ala263 | (5*S*, 6*S*)-S-TeA C10  (5*S*, 6*S*)-S-TeA C8  **(5*S*, 6*S*)-S-TeA O2**  (5*S*, 6*S*)-S-TeA C7  (5*S*, 6*S*)-S-TeA C12  (5*S*, 6*S*)-S-TeA C9 | Alkyl hydrophobic  Pi hydrophobic  **Hydrogen bond**  Pi hydrophobic  Alkyl hydrophobic  Alkyl hydrophobic | 3.32  3.42  **3.09**  3.44  3.37  3.42 | −34.06 |
| (5*S*, 6*R*)-S-TeA | 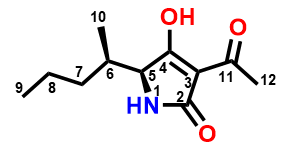 | 392.13 | D1-Val249  D1-His252  D1-Tyr254  **D1-Gly256 NH** | (5*S*, 6*R*)-S-TeA C12  (5*S*, 6*R*)-S-TeA C10  (5*S*, 6*R*)-S-TeA C12  **(5*S*, 6*R*)-S-TeA** **O2** | Alkyl hydrophobic  Alkyl hydrophobic  Alkyl hydrophobic  **Hydrogen bond** | 3.31  3.64  3.56  **3.72** | −27.32 |
| (5*R*, 6*S*)-S-TeA | 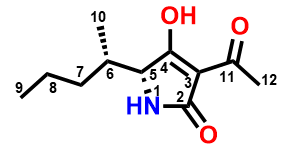 | 389.91 | D1-Val249  D1-Gly253 | (5*R*, 6*S*)-S-TeA C10  (5*R*, 6*S*)-S-TeA C9 | Alkyl hydrophobic  Alkyl hydrophobic | 3.47  3.81 | −15.23 |
| (5*R*, 6*R*)-S-TeA | 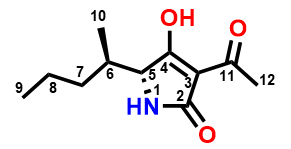 | 388.72 | — | — | — | — | −8.66 |
