# Supplementary material for: Discovery, Herbicidal Activity and Biosynthesis of a Novel Natural Tetramic Acid from Alternaria Species
Source: Adv Sci (Weinh). 2025 Apr 25;12(21):2416188. doi: 10.1002/advs.202416188 (PMC12140367; doi:10.1002/advs.202416188)
Supplement: Supplementary file 9 — Supplemental Table 8 [file ADVS-12-2416188-s003.docx]

**Table S8.** Structure and possible interactions for isoleucine (Ile) and 2-amino-3-methylhexanoic acid (AMHA) binding to the *Aa*TAS1 protein.

| Compound | Chemical structure | Bonding donors | Bonding acceptors | Interactions | Bound distance (Å) | Interaction energy  (kcal mol^−1^) |
| --- | --- | --- | --- | --- | --- | --- |
| Ile |  | *Aa*TAS1-Arg544  *Aa*TAS1-Met564  *Aa*TAS1-Arg567  ***Aa*TAS1-Thr678 OH** | Ile C5  Ile C5  Ile C6  Ile **CO** | Alkyl hydrophobic  Alkyl hydrophobic  Alkyl hydrophobic  **Hydrogen bound** | 3.35  3.15  2.86  **2.25** | −36.59 |
| AMHA |  | *Aa*TAS1-Arg544  *Aa*TAS1-Met564  *Aa*TAS1-Asp566  *Aa*TAS1-Arg567  ***Aa*TAS1-Thr678 OH** | AMHA C6  AMHA C6  AMHA C7  AMHA C7  AMHA **CO** | Alkyl hydrophobic  Alkyl hydrophobic  Alkyl hydrophobic  Alkyl hydrophobic  **Hydrogen bound** | 3.36  3.13  3.05  2.87  **2.26** | −36.57 |
